# Synaptopathology Involved in Autism Spectrum Disorder

**DOI:** 10.3389/fncel.2018.00470

**Published:** 2018-12-21

**Authors:** Shiqi Guang, Nan Pang, Xiaolu Deng, Lifen Yang, Fang He, Liwen Wu, Chen Chen, Fei Yin, Jing Peng

**Affiliations:** ^1^Department of Pediatrics, Xiangya Hospital, Central South University, Changsha, China; ^2^Hunan Intellectual and Developmental Disabilities Research Center, Changsha, China

**Keywords:** autism, ASD, synapse, synaptogenesis, synapse elimination, synaptic transmission, synaptic plasticity

## Abstract

Autism spectrum disorder (ASD) encompasses a group of multifactorial neurodevelopmental disorders characterized by impaired social communication, social interaction and repetitive behaviors. ASD affects 1 in 59 children, and is about 4 times more common among boys than among girls. Strong genetic components, together with environmental factors in the early stage of development, contribute to the pathogenesis of ASD. Multiple studies have revealed that mutations in genes like *NRXN, NLGN*, *SHANK*, *TSC1/2*, *FMR1*, and *MECP2* converge on common cellular pathways that intersect at synapses. These genes encode cell adhesion molecules, scaffolding proteins and proteins involved in synaptic transcription, protein synthesis and degradation, affecting various aspects of synapses including synapse formation and elimination, synaptic transmission and plasticity. This suggests that the pathogenesis of ASD may, at least in part, be attributed to synaptic dysfunction. In this article, we will review major genes and signaling pathways implicated in synaptic abnormalities underlying ASD, and discuss molecular, cellular and functional studies of ASD experimental models.

## Introduction

The term “autism” as a unique disease concept was first used by Leo Kanner in 1943. He described eight boys and three girls, aged two to eight, who were unable to use language to communicate, displaying monotonous repetitions and preoccupation with objects (Kanner, 1995). Then in 1944, Hans Asperger identified a group of children who could not integrate into their social environment and showed certain stereotypic behaviors, but did not have the speech deficiency of typical autism (Hippler and Klicpera, 2003). In recent years, the term autism spectrum disorder (ASD) has gained general acceptance to describe a set of multifactorial neurodevelopmental disorders, grouping disorders including autistic disorder, Asperger Syndrome, pervasive developmental disorder-not otherwise specified (PDD-NOS) and child disintegrative disorder under the umbrella ASD. Although ASD has diverse behavioral manifestations with varying degrees of severity, it has a core dyad of impairments: (1) impaired social communication and social interaction, and (2) restricted and repetitive behavior, interests or activities. There are also a number of comorbidities associated with ASD, including intellectual disability (ID), epilepsy (Tuchman et al., 2010), anxiety, attention deficits hyperactivity disorder (ADHD), sleeping disorders, and gastrointestinal disorders (Doshi-Velez et al., 2014). ASD affects 1 in 59 children in the United States, and is about 4 times more common among boys than among girls according to the estimate from CDC’s Autism and Developmental Disabilities Monitoring (ADDM) Network (Baio et al., 2018). Large-scale surveys show the median worldwide prevalence of ASD is 1–2% (Kim et al., 2011; Blumberg et al., 2013). ASD represents a lifelong condition for patients and many of them require special educational and social support, so ASD imposes a huge financial and emotional burden on society (Lavelle et al., 2014; Leigh and Du, 2015). However, only limited treatment of ASD is available, in part because the causes of ASD have not yet been clearly elucidated.

Based on current studies, strong genetic components contribute to the pathogenesis of ASD, together with environmental factors in the early stage of development. Early twin studies showed that concordance of autism in monozygotic (MZ) twins (80–90%) was much higher than that in dizygotic (DZ) twins (0–10%) (Folstein and Rutter, 1977; Ritvo et al., 1985; Steffenburg et al., 1989; Bailey et al., 1995). However, these early studies have their limitations because of their small sample size, restricted follow-up time, and vicissitudes of diagnostic criteria. In recent years, multiple population-based cohort studies provide more reliable evidence for ASD genetic predisposition. The relative recurrence rate for MZ twins (153.0) is much higher than that for DZ twins (8.2) (Sandin et al., 2014) and estimates of the heritability of ASD range from 54% to 95% (Gaugler et al., 2014; Sandin et al., 2014; Colvert et al., 2015). In addition to genetic predisposition, known environmental risk factors of ASD include advancing parental (especially paternal) reproductive age (Gardener et al., 2009; Hultman et al., 2011; Sandin et al., 2012), complications during pregnancy (Brown et al., 2014; Estes and McAllister, 2016) and prenatal exposure to psychotropic drugs like valproic acid (VPA) (Rasalam et al., 2005; Meador et al., 2013; Boukhris et al., 2016).

Recent advances in gene testing techniques have allowed identification of more than 1,000 candidate genes and copy number variants (CNVs) loci associated with ASD, according to the Simmons Foundation Autism Research Initiative (SFARI) gene database^1^. It is estimated that a genetic cause can be identified in up to 25% of ASD cases, chromosomal rearrangements (encompassing rare and de novo CNVs) and coding-sequence mutations making up ∼10–20% and ∼5–10% of ASD patients, respectively (Huguet et al., 2013; Ziats and Rennert, 2016). Numerous ASD candidate genes implicated in many aspects of basic cell function such as chromatin remodeling, metabolism, mRNA translation may impact neuronal processes ranging from neurogenesis to neuron migration, axon guidance, dendrite outgrowth, and synaptic formation and function (Gilbert and Man, 2017).

Synapses are highly specialized asymmetric cell-cell junctions that are fundamental units of brain communication. ASD is diagnosed at an early stage of life, usually before three years of age, a period when intense synaptogenesis is happening (Huttenlocher and Dabholkar, 1997). Multiple studies have found that mutations in genes like *NRXN, NLGN*, *SHANK*, *TSC1/2*, *FMR1*, and *MECP2* (Table 1) converge on common cellular pathways that converge at the synapse (Wang T. et al., 2016; Stessman et al., 2017). ASD patients are found to carry a higher global burden of rare, large CNVs which can include known ASD associated synaptic genes, such as *SHANK3* and *CHD10* (Guo et al., 2017). These genes encode cell adhesion molecules, scaffolding proteins, and proteins involved in synaptic transcription, protein synthesis and degradation, which affect various aspects of synapses, including synapse formation and elimination, synaptic transmission and plasticity, suggesting that the pathogenesis of ASD, at least in part, may be attributed to synaptic dysfunction, which can lead to functional and cognitive impairments. Some environmental risk factors of ASD can also bring about synaptic defects. For instance, the patterns of long-term potentiation (LTP) of mice that are prenatally exposed to VPA pass from an enhanced LTP phenotype in early life to an impaired LTP phenotype in adulthood (Rinaldi et al., 2007). Synaptic impairments caused by gene mutations and environmental factors are implicated in many other neurodevelopmental diseases including epilepsy, ID, developmental delay, and attention deficit-hyperactivity disorder (ADHD). Some neuropsychiatric disorders, including schizophrenia (SCZ), bipolar disorder and obsessive-compulsive disorder (OCD) are also associated with synaptic dysfunction. Though rare non-syndromic gene mutations only account for a small fraction of ASD cases, they provide an excellent window to better understanding the synaptic abnormalities underlying ASD. Insight has also been gained from studies of the synaptopathology of monogenic diseases such as fragile X syndrome, tuberous sclerosis complex, and Angelman syndrome that share a high comorbidity with ASD. In this article, we will review abnormalities in synaptogenesis, synaptic elimination, synaptic transmission and plasticity that are caused by mutations of a number of ASD-associated genes, to gain a better understanding of the synaptic dysfunctions underlying ASD.

**Table 1:** Critical ASD candidate genes and major molecular functions in synapse.

	Gene symbol	Chromosomal Locus	Protein	Major molecular functions in synapses	SFARI score
Cell adhesion molecules	*NRXN1*	2p16.3	Neurexin 1	Synaptogenesis, synaptic transmission, synaptic plasticity	2
	*NRXN2*	11q13.1	Neurexin 2		4
	*NRXN3*	14q24.3-q31.1	Neurexin 3		3
	*NLGN1*	3q26.31	Neuroligin 1		3
	*NLGN2*	17p13.1	Neuroligin 2		4
	*NLGN3*	Xq13.1	Neuroligin 3		2
	*NLGN4*	Xp22.32-p22.31	Neuroligin 4		3
	*NLGN4Y*	Yq11.221	Neuroligin 4Y		4
	*CNTNAP2*	7q35-q36.1	contactin associated protein-like 2	Synaptogenesis, synaptic transmission	2/S
	*CDH8*	16q21	Cadherin 8	Synaptogenesis, synaptic transmission	4
	*CDH11*	16q21	Cadherin 11		4
	*CNTN4*	3p26.3-p26.2	Contactin 4	Synaptogenesis	2
	*CNTN6*	3p26.3	Contactin 6		3
	*PCDH10*	4q28.3	Protocadherin 10	Synaptic elimination	4
Scaffolding proteins	*DLG4*	17p13.1	discs large MAGUK scaffold protein 4	Synaptogenesis, synaptic elimination, synaptic plasticity	5
	*GPHN*	14q23.3-24.1	Gephyrin	Synaptogenesis	3
	*SHANK1*	19q13.33	Shank 1	Synaptogenesis, synaptic transmission, synaptic plasticity	3
	*SHANK2*	11q13.3-q13.4	Shank 2		2
	*SHANK3*	22q13.33	Shank 3		1/S
	*HOMER1*	5q14.1	Homer homolog 1	Synaptogenesis, synaptic plasticity	4
Regulators of protein synthesis	*FMR1*	Xq27.3	Fragile mental retardation protein	Synaptogenesis, synaptic transmission, synaptic elimination, synaptic plasticity	S
and degradation	*CYFIP1*	15q11.2	cytoplasmic FMR1 interacting protein 1	Synaptogenesis, synaptic transmission, synaptic plasticity	3
	*TSC1*	9q34.13	tuberous sclerosis 1	Synaptogenesis, synaptic plasticity	S
	*TSC2*	16p13.3	tuberous sclerosis 2	Synaptogenesis, synaptic plasticity	3/S
	*EIF4E*	4q23	eukaryotic translation initiation factor 4E	Synaptogenesis, synaptic transmission, synaptic plasticity	4
	*EIF4EBP2*	10q22.1	Eukaryotic translation initiation factor 4E binding protein 2	Synaptogenesis, synaptic transmission, synaptic plasticity	5
	*PTEN*	10q23.32	phosphatase and tensin homolog	Synaptogenesis, synaptic plasticity	1/S
	*SYNGAP1*	6p21.32	synaptic Ras GTPase activating protein 1	Synaptogenesis, synaptic plasticity	1/S
	*UBE3A*	15q11.2	ubiquitin protein ligase E3A	Synaptogenesis, synaptic transmission, synaptic elimination	3/S
Regulators of chromatin	*MECP2*	Xq28	methyl CpG binding protein 2	Synaptogenesis, synaptic plasticity	2/S
remodeling and transcription	*MEF2C*	5q14.3	myocyte enhancer factor 2C	Synaptic elimination	4/S


*The SFARI score is used to evaluate the strength of association between candidate gene and ASD. (1) high confidence; (2) strong candidate; (3) suggestive evidence; (4) minimal evidence; (5) hypothesized but untested. S-syndromic genes leading to syndromes that share high comorbidity with ASD.*

## Cell Adhesion Molecules

### Neurexin/Neuroligin Complex

Neurexins (Nrxn) are a family of presynaptic transmembrane proteins encoded by paralogous genes, *NRXN1, NRXN2, NRXN3*. Each of the three genes uses 2 independent promotors to generate 2 major protein isoforms, a long α-Nrxn and a short β-Nrxn. Loss-of-function variants in *NRXN1* have been identified in patients with ASD in multiple cohort studies (Marshall et al., 2008; Morrow et al., 2008; Glessner et al., 2009). A wide spectrum of developmental disorders including ID without ASD (Camacho-Garcia et al., 2012; Yangngam et al., 2014), language delay, mild dysmorphic features, and hypotonia (Ching et al., 2010), as well as neuropsychiatric conditions like SCZ have also been reported in patients with *NRXN1* variants. In contrast, variants in *NRXN2* and *NRXN3* are much rarer, but accumulating reports provide evidence supporting an association between these two genes and ASD, as well as other neurodevelopmental and neuropsychiatric conditions (Gauthier et al., 2011; Vaags et al., 2012; Yuan et al., 2018).

Neuroligins (Nlgns), encoded by five genes (*NLGN1*, *NLGN2*, *NLGN3*, *NLGN4*, and *NLGN4Y*) in the human genome, are a class of cell-adhesion molecules on the postsynaptic membranes, binding to the laminin/neurexin/sex hormone (LNS) binding domain of Nrxn through the extracellular cholinesterase-like domain. Nlgn1 is expressed exclusively on excitatory synapses and binds to PSD-95, whereas Nlgn2 is restricted to inhibitory synapses, preferentially binding to gephyrin (Song et al., 1999; Varoqueaux et al., 2004). Nlgn3 and Nlgn4 are present on both types of synapses (Budreck and Scheiffele, 2007).

Nrxn binds to its postsynaptic partner Nlgn to form a Ca^2+^-dependent neurexin/neuroligin complex, implicated in synaptogenesis and required for efficient neurotransmission. After the initial contact of an axon with a target cell, Nrxn and Nlgn together recruit key synaptic components including neurotransmitter receptors and scaffolding proteins to promote synaptic assembly, maturation and differentiation (Krueger et al., 2012; Reissner et al., 2013; Sudhof, 2017). Overexpression of Nlgn or Nrxn can increase the number of synapses, leading to an alteration in the excitatory/inhibitory (E/I) ratio (Chih et al., 2004; Chubykin et al., 2007; Dahlhaus et al., 2010). E/I balance is necessary for appropriate circuit development and synaptic plasticity, and its alteration impairs information processing and social function, contributing to the pathogenesis of ASD and many other neurodevelopmental diseases (Gatto and Broadie, 2010; Nelson and Valakh, 2015). In *Drosophila*, Nlgn1 directly interacts with WAVE regulatory complex (WRC) and recruits WRC to postsynaptic sites to reorganize postsynaptic actin, thus maintaining normal synaptic formation and transmission in the *Drosophila* neuromuscular junction (NMJ) (Xing et al., 2018). Nlgn1 exerts its effect on differentiation and LTP of excitatory synapses via phosphorylation of intracellular tyrosine, which affects the recruitment of AMPARs (Letellier et al., 2018).

Nrxn- or Nlgn-deficient neurons display aberrant synaptic function, but not significant changes in the formation of synapses. Nrxn -1α deficient neurons show a selective reduction in the excitatory synaptic strength reflecting a decreased neurotransmitter release probability, leading to E/I imbalance (Etherton et al., 2009; Pak et al., 2015). Nrxn-1α acts as a positive regulator of Ca^2+^ influx through α2δ-1 subunits that are responsible for trafficking and kinetic properties of Ca_V_2.1 pore-forming subunits, ensuring proper vesicle release (Brockhaus et al., 2018). Moreover, *NRXN1*-mutant human neurons exhibit increased levels of CASK, a scaffolding protein binding to Nrxn1, and mutations in *CASK* are associated with ASD, brain malformation, mental retardation (Najm et al., 2008; Hackett et al., 2010). Mice lacking Nrxn-2α display deficits in social behaviors and increased anxiety-like behaviors, accompanied by impaired glutamatergic release in the neocortex (Dachtler et al., 2014, 2015; Born et al., 2015). Mice with combined deletion of Nrxn-2α and Nrxn2β show defects of excitatory release similar to mice lacking Nrxn-2α, indicating that Nrxn-2β has no strong function in basic transmission (Born et al., 2015). In addition, there is a significant decrease of Munc18-1 in the hippocampus of mice lacking Nrxn-2α, suggesting deficiencies in presynaptic vesicular release (Dachtler et al., 2014). Munc18-1 is involved in synaptic vesicle docking, priming and fusion (Rizo and Xu, 2015), encoded by *STXBP1*, which is also associated with ASD (Wang T. et al., 2016; Uddin et al., 2017). The cytoplasmic tail of Nrxn captures Munc18 via a multiprotein complex with involvement of Mint1 to facilitate presynaptic vesicular release (Biederer and Sudhof, 2000).

Triple knockout mice lacking Nlgn1-3 die shortly after birth due to severely impaired synaptic transmission of both excitatory and inhibitory synapses, but the density of synaptic contacts is not altered (Varoqueaux et al., 2006). Mice with Nlgn1 deletion show reduced NMDA/AMPR ratio in the striatum, accounting for a dramatic increase in repetitive, stereotyped grooming behavior, and the NMDAR partial co-agonist D-cycloserine can rescue this phenotype (Blundell et al., 2010). Mice with R451C substitution in Nlgn3 show an increased AMPAR- and NMDAR-mediated synaptic transmission and enhanced LTP in the hippocampus (possibly explaining the observed enhanced spatial learning abilities), but in the somatosensory cortex, inhibitory synaptic transmission is increased without apparent alteration in excitatory transmission (Tabuchi et al., 2007; Etherton et al., 2011a; Jaramillo et al., 2014). R451C substitution causes retention of Nlgn3 in the endoplasmic reticulum, resulting in decreasing delivery of Nlgn3 to the postsynaptic membranes (Comoletti et al., 2004). Strangely, mice with deletion of Nlgn3 do not exhibit any of these change (Tabuchi et al., 2007; Etherton et al., 2011b). It has been shown that knockout of *Nlgn3* late in development produces a marked synaptic phenotype, but no detectable effects are seen after knockout during early development, apparently due to developmental compensation by cerebellin-1 (Clbn1), a secreted protein binding to Nrxn (Xing et al., 2018). This may explain why *Nlgn3* KO mice have no phenotype in previous studies. R704C substitution in Nlgn3 results in a major and selective decrease in AMPAR-mediated synaptic transmission in pyramidal neurons of hippocampus, without altering NMDAR- and GABAR-mediated synaptic transmission, as well as presynaptic neurotransmitter release (Etherton et al., 2011b). *Nlgn4* KO significantly decreases the peak amplitude and the frequency of spontaneous inhibitory postsynaptic currents (sIPSCs), without changing mIPSCs, sEPSCs and mEPSCs in CA3 pyramidal cells, confirming that Nlgn4 knockout affects primarily inhibitory transmission (Hammer et al., 2015).

## Scaffolding Proteins

### 2.1 PSD-95

PSD-95, encoded by the *DLG4* gene, is the central element of postsynaptic architecture of excitatory synapses. Rare missense mutations and CNVs in *DLG4* may increase susceptibility for ASD and SCZ (Roberts et al., 2014; Wang J. et al., 2016; Xing et al., 2016). Recently, truncation variants in *DLG4* have been reported to be responsible for ID with marfanoid features (Moutton et al., 2018). Through its three PDZ domains, an SH domain and a guanylate kinase homology (GK) domain, PSD-95 potentially binds to many key constituent PSD proteins, including AMPARs via Stargazin/TARP, NMDARs, adhesion molecules like Nlgn1, and other scaffolding proteins, such as GKAP. The binding diversity suggests that it is of critical importance for synaptic structural organization (Feng and Zhang, 2009). Overexpression of PSD-95 shifts the distribution of Nlgn2 from inhibitory synapses to excitatory synapses, resulting in a decrease in the total number of inhibitory synapses, demonstrating that levels of PSD-95 can manipulate the E/I balance (Levinson et al., 2005). RNAi knockdown of PSD-95 results in shortening and thinning of PSD and loss of putative AMPAR-type structures, confirming that PSD-95 is essential for sustaining molecular organization of PSD (Chen et al., 2011). Time-lapse imaging of axon-dendrite contacts shows that overexpression of PSD-95 reduces the turnover rates of pre- and postsynaptic structures, thus promoting the stabilization of putative synaptic contacts (Taft and Turrigiano, 2014).

PSD-95 exerts a strong influence on synaptic strength through its role in controlling neurotransmitter receptors. Complete genetic deletion of PSD-95 leads to the reduction in the number of AMPARs in synapses, forming silent synapses that contain a high abundance of NMDARs but no AMPARs. This bears important consequences for enhanced LTP, because during LTP expression, synaptic strength increases due to the incorporation of AMPARs into postsynaptic membranes (Béïque et al., 2006; Huang et al., 2015). In addition, PSD-95 downregulation increases the clustering of NR2B-containing NMDARs, which are characterized by slow kinetics and long decay time, resulting in a larger flow of Ca^2+^ (Béïque et al., 2006; Bustos et al., 2014). NR2B subunit plays an essential role in the synaptic maturation and working memory functions, but its overabundance may lead to increased susceptibility to neuroexcitotoxicity (Monaco et al., 2015).

### Gephyrin

Gephyrin, encoded by *GPHN* gene, is a key scaffolding protein at the inhibitory synapses, comprising three functional domains, G-, C- and E-domain. Rare de novo or inherited microdeletions and point mutations of *GPHN* have been reported in the patients with ASD, seizures and epileptic encephalopathy (Lionel et al., 2013; Dejanovic et al., 2014a, 2015; Hu et al., 2015). Cohort studies with large sample size add to the growing evidence that CNVs involving *GPHN* contribute to ASD (Prasad et al., 2012; Egger et al., 2014). Gephyrin directly interacts with β-subunit of glycine receptors (GlyR) and α-subunits of GABA_A_ receptor (GABA_A_R) via its E-domain. Nlgn2 preferentially binds to gephyrin through a conserved tyrosine residue to activate the gephyrin-interacting protein collybistin, a neuron-specific GDP/GTP exchange factor. Gephyrin thus acts as a molecular hub to promote the clustering of inhibitory neurotransmitter receptors and regulate the formation and extension of the inhibitory postsynaptic specialization.

Unlike their counterparts at glutamatergic synapses, gephyrin and its binding partners lack canonical protein interaction motifs, suggesting that they rely on different regulation mechanisms such as posttranslational modification. Gephyrin monomers oligomerize to form a hexagonal lattice of gephyrin clusters underneath the cell membrane that slow the lateral mobility of GlyRs and GABA_A_Rs, thus allowing their clustering at postsynaptic sites (Groeneweg et al., 2018). Phosphorylation of gephyrin on S270 by GSK-3β, on S268 by ERK and on S305 by CaMKII modulate the density and size of gephyrin clusters, thus affecting GABAergic transmission and homeostatic synaptic plasticity (Tyagarajan et al., 2011, 2013; Flores et al., 2015). It has been shown that small ubiquitin-like modifier (SUMO)-ylation acts upstream of phosphorylation on S268 of gephyrin (Ghosh et al., 2016). Palmitoylation of gephyrin, found to be GABA_A_R activity-dependent, is crucial for the association of gephyrin with postsynaptic membranes and for gephyrin clustering, and also strengthens GABAergic synaptic transmission (Dejanovic et al., 2014b). Inhibition of nitric oxide synthase (nNOS) results in a loss of S-nitrosylation of gephyrin and the formation of larger gephyrin clusters, ultimately increasing the number of GABA_A_Rs on the postsynaptic membranes (Dejanovic and Schwarz, 2014). PKC-dependent phosphorylation and calcineurin-dependent dephosphorylation of growth-associated protein 43 (GAP-43), an activity-dependent phosphoprotein mediating neurite growth, are also related to gephyrin folding and clustering (Wang et al., 2015). Expression of chemically induced LTP of inhibition (iLTP) requires synaptic recruitment of gephyrin from extrasynaptic areas to immobilize and confine GABA_A_Rs at synapses, which in turn is promoted by CaMKII-dependent phosphorylation of GABA_A_R-β3-Ser383 (Petrini et al., 2014).

### Shank

Shank (also known as ProSAP) proteins consist of three major isoforms, Shank1, Shank2, and Shank3, which have similar sets of domains: N-terminal ankyrin repeats, a Src homology 3 (SH3) domain, a PSD-95/Discs large/ZO-1 (PDZ) domain, a long proline-rich region, and a sterile alpha motif (SAM) domain. As master scaffolding proteins at the PSD of excitatory synapses, Shank proteins interact with more than 30 synaptic proteins through these multiple domains, essential for synaptic formation, glutamate receptor trafficking and neuronal signaling (Monteiro and Feng, 2017). In *Drosophila*, both deletion and overexpression of Shank result in defects in synaptic bouton number and maturation via regulating the internalization of the Wnt receptor Frizzled-2 (Fz2) (Harris et al., 2016).

Shank3 is encoded by *SHANK3*, located on chromosome 22q13.3. *SHANK3* was first identified in the 22q13.3 deletion syndrome, also known as Phelan-McDermid Syndrome (PMS), which is characterized by global developmental delay (Phelan et al., 2001; Phelan and McDermid, 2012). More than 50% of the patients with PMS show autism and autistic-like behaviors. *SHANK3* variants including de novo deletions, insertion, splicing mutations and point mutations have been identified in ASD patients (Durand et al., 2007; Gauthier et al., 2009; Boccuto et al., 2013). Prevalence of *SHANK3* variants in patients with ASD and ID is estimated to 1–2%, whereas *SHANK1* and *SHANK2* mutations are much rarer (Boccuto et al., 2013; Leblond et al., 2014). Shank3-deficient neurons show reduced levels of PSD proteins including GKAP, Homer1b/c, AMPAR subunit GluA1 and NMDAR subunit NR2A (Peca et al., 2011; Wang et al., 2011). Disrupted interactions among Shank3, GKAP and Homer1b/c may account for the impaired activity-dependent redistribution of the GluA1 subunit, leading to reduced post-tetanic potentiation and hippocampal LTP (Wang et al., 2011). Neurons induced from pluripotent stem cells (iPSCs) in patients with PMS show significantly impaired NMDAR- and AMPAR-mediated synaptic transmission (Shcheglovitov et al., 2013). Shank3 deficient mice display self-injurious repetitive grooming and deficits in social interaction with significant reductions in frequency and peak amplitude of mEPSCs, which suggests a decreased number of functional synapses and reduced postsynaptic response from the available synapses, respectively (Peca et al., 2011). In contrast, overexpression of Shank3 leads to a dramatic increase in the amplitude of evoked AMPAR- and NMDAR-mediated EPSCs, as well as higher frequency of AMPAR-mediated mEPSCs (Arons et al., 2012).

Shank2 is encoded by *SHANK2*, located on chromosome 11q13.3-q13.4. *SHANK2* variations including deletions, missense mutations, truncations, and mutations in the *SHANK2* promoter regions have been identified, linking *SHANK2* variants to ASD and ID (Berkel et al., 2010). Single nucleotide polymorphisms (SNPs) in *SHANK2* introns are also implicated in ASD risk (Bai et al., 2018). Discrepant effects on synaptic transmission and long-term plasticity are reported in two independently generated *Shank2* KO mice, *Shank2* Δ*e7^–/–^* and *Shank2 e6-7^–/–^*. *Shank2* Δ*e7^–/–^* mice (Schmeisser et al., 2012) display extreme hyperactivity and profound autism-like behavior, such as repetitive grooming and abnormalities in vocal and social behaviors, accompanied by reduced basal synaptic transmission, decreased frequency of mEPSCs and an increased NMDA/AMPA ratio consistent with increased levels of the NMDAR subunit GluN1 in the hippocampus and striatum. LTP of Schaffer collaterals is slightly enhanced in *Shank2* Δ*e7^–/–^* mice, but no evidence for alterations in LTD. Recently, Wegener et al. (2018) reproduced these results in *Shank2* Δ*e7^–/–^* mice and further uncovered a developmental synapse phenotype, an excess of silent synapses, which explains the phenomena of decreased synaptic transmission and enhanced LTP. *Shank2* Δ*e6-7^–/–^* mice (Won et al., 2012) exhibit hyperactivity and autism-like behavior too, though with a reduced NMDA/AMPA ratio and selectively decreased NMDAR-mediated synaptic transmission, as well as severely impaired LTP and LTD. Direct stimulation of NMDARs with D-cycloserine (a partial agonist of NMDARs), and a positive allosteric modulator of metabotropic glutamate receptor 5 (mGluR5) that enhances NMDAR function via mGluR5 activation can normalize NMDAR function and improves social interaction of *Shank2* Δ*e6-7^–/–^* mice. Mice with conditional *Shank2* deletion (exon 6–7) restricted to parvalbumin (PV)-positive neurons display moderate hyperactivity and enhanced self-grooming, suggesting that *Shank2* deletion in PV-positive neurons contributes to the hyperactivity observed in global *Shank2*-KO mice (Lee et al., 2018). Interestingly, expression levels of *Gabra2* and GABA_A_R-mediated inhibitory synaptic transmission are reduced in *Shank2* Δ*e6-7^–/–^* mice, but not in *Shank2* Δ*e7^–/–^* mice, and enhancing inhibitory synaptic function with an allosteric modulator for the GABAA Rs reverses spatial memory deficits in *Shank2* Δ*e6-7^–/–^* mice (Lim et al., 2017).

Shank1 is encoded by *SHANK1*, located on chromosome 19q13.33. Deletions and point mutations of *SHANK1* have been identified in ASD individuals, indicating the involvement of *SHANK1* in ASD (Sato et al., 2012). Mice completely lacking all Shank1 protein isoforms (*Shank1^–/–^* mice) have increased anxiety-related behavior, impaired contextual fear memory and object recognition memory (Hung et al., 2008; Sungur et al., 2017), as well as social communication/interaction deficits (Wohr et al., 2011; Sungur et al., 2016). Adult, but not juvenile *Shank^+/-^* heterozygous and *Shank1^–/–^* null mutant mice displayed slightly elevated levels of self-grooming behavior (Sungur et al., 2014). Silverman et al. (2011) found that motor functions were reduced on open field activity, rotarod, and wire hang in *Shank1^–/–^* mutants, extending the role of Shank1 to motor functions. *Shank1^–/–^* mice show smaller dendritic spines, thinner and smaller PSD, and altered PSD protein composition, as well as weaker basal synaptic transmission, affirming the importance of Shank1 for synaptic structure and function *in vivo* (Hung et al., 2008). Shank1 is highly expressed in parvalbumin-expressing (PV+) fast-spiking inhibitory interneurons in hippocampus, and loss of Shank1 causes increased E/I ratio by weakening inhibitory synaptic function, which is consistent with decreased expression of gephyrin (Mao et al., 2015).

## Synaptic Gene Transcription, Protein Synthesis and Degradation

It is well accepted that a stable proteome is important for synaptic plasticity, and dysregulation of synaptic protein synthesis, recycling, and degradation at the activated synapse is implicated in the pathogenesis of neurological diseases including ASD, ID and epilepsy (Cajigas et al., 2010; Klein et al., 2016; Louros and Osterweil, 2016). Of special interest is the phosphoinositide 3-kinase /AKT/mammalian target of rapamycin (PI3K/AKT/mTOR) signaling pathway, which acts as a central regulator of a diverse array of cellular processes, including cell growth, proliferation and protein translation. Components of the mTOR pathway are present at synapses, where they play important roles in synaptogenesis, synaptic transmission and plasticity.

### TSC1/2

Hamartin (TSC1) and tuberin (TSC2) are protein products of the *TSC1* and *TSC2* genes, respectively. These two proteins together with the recently identified protein TBC1D7 form a complex that inhibits the activity of mTOR complex 1 (mTORC1) through the inhibition of Ras homolog expressed in brain (Rheb), a GTPase located upstream of mTORC1. Spontaneous or inherited mutations in *TSC1* and *TSC2* genes lead to dysfunction of the TSC1/TSC2/TBC1D7 complex and disinhibition of mTORC1, thus resulting in tuberous sclerosis complex (TSC). TSC is a neurocutaneous disorder characterized by the formation of hamartomas in multiple organs, and neurological manifestations including epilepsy, ID and ASD. ASD is estimated to affect about 40% of patients with TSC (Richards et al., 2015), and epilepsy is one of the risk factors for the development of ASD in TSC patients (Vignoli et al., 2015; Mitchell et al., 2017).

Both heterozygous and homozygous loss of *Tsc1* in mouse cerebellar Purkinje cells (PCs) result in autistic-like behaviors, including abnormal social interaction, repetitive behavior, and vocalizations (Tsai P.T. et al., 2012). PCs in the model mouse show significantly reduced spontaneous EPSC (sEPSC) frequency and mEPSC frequency, indicating a decreased number of functional glutamatergic synapses; importantly, human neurons derived from TSC2-deficient iPSCs display the same defects (Costa et al., 2016; Sundberg et al., 2018). The expression levels of the PC-specific glutamate receptor δ2 (GRID2), the postsynaptic marker PSD-95, and presynaptic marker synaptophysin (SYP1) are all reduced, suggesting abnormal synaptic development (Sundberg et al., 2018). High-throughput RNA sequencing reveals a down-regulation of genes associated with neurogenesis and glutamate receptor signaling in cortical tubers of TSC patients (Mills et al., 2017).

Eker rats, which carry a spontaneous *Tsc2* germline mutation, exhibit marked reduction in LTP and LTD elicited by different protocols in hippocampal synapses (von der Brelie et al., 2006). Mice with a heterozygous mutation of *Tsc2* show a reduced threshold for late-phase of LTP in the CA1 region of hippocampus, which can lead to inappropriate storage of unrelated or unprocessed information, thus impairing hippocampal-dependent learning (Ehninger et al., 2008). Mutations in both *TSC1* and *TSC2* abolish metabotropic glutamate receptor-dependent LTD (mGluR-LTD) in hippocampal CA1 synapses, without affecting NMDAR-mediating LTD (Auerbach et al., 2011; Bateup et al., 2011). Inhibition by negative allosteric modulator (NAM) of mGluR5 corrects hyperactivity, seizures and elevated synaptic protein synthesis in *Tsc2* mutant mice, whereas positive allosteric modulation of mGluR5 results in the exacerbation of hyperactivity and epileptic phenotypes, suggesting a meaningful therapeutic potential for mGluR5 NAM in TSC (Kelly et al., 2018).

### PTEN

PTEN (phosphatase and tensin homolog), encoded by the *PTEN* gene on chromosome 10q23, is a dual lipid/protein phosphatase, best known for its role in tumor suppression. PTEN dephosphorylates phosphatidylinositol 3,4,5-trisphosphate (PIP3) to generate the bisphosphate product phosphatidylinositol 4,5-bisphosphate (PIP2), further negatively regulating the PI3K/AKT/mTOR signaling pathway, ultimately inhibiting cellular survival and proliferation. PTEN is also highly expressed in the central nervous system, where it is intimately involved in neuronal growth and synaptic function. Loss-of-function mutations in *PTEN* contribute to macrocephaly, seizures, ASD and mental retardation (Butler et al., 2005; Buxbaum et al., 2007; Orrico et al., 2009; Varga et al., 2009), and incomplete loss of *PTEN* function is more commonly linked to ASD (Spinelli et al., 2015). *PTEN* mutations are found in 7–27% of macrocephalic ASD individuals and 8% of macrocephalic ID patients (McBride et al., 2010; Hobert et al., 2014).

Conditional knockout (cKO) mice in which Pten is ablated in granule cells of dentate gyrus (DGC) were reported to show increased evoked synaptic transmission in both glutamatergic and GABAergic neurons, resulting from an increase in the number of synaptic vesicles available for release and upregulated synaptogenesis (Weston et al., 2012). However, others studying Pten-deleted neurons reported increased excitatory synaptic currents but unchanged IPSC amplitude, thus shifting the E/I balance, leading to hyperactivity of mutant neurons (Luikart et al., 2011; Williams et al., 2015). cKO of *Pten* in PCs results in autistic-like traits in adult *Pten*-mutant mice, and mutant PCs show structural abnormalities in dendrites and axons and decreased excitability, as well as altered excitatory postsynaptic currents (Cupolillo et al., 2016). Although loss of *PTEN* during early development is associated with substantial structural abnormalities, it has been shown that the effects of *PTEN* on neuronal morphology and synaptic function are independent of each other. cKO mice with Pten deletion restricted to forebrain excitatory neurons displayed impaired LTP and LTD at excitatory hippocampal synapses without any changes in morphology, which coincides with impaired spatial memory (Sperow et al., 2012). Pten-deleted DGC synapses showed enhanced theta burst-induced LTP and impaired mGluR-LTD prior to the onset of gross morphological defects (Takeuchi et al., 2013). PTEN can modulate synaptic activity via a PDZ-dependent association between PTEN and PSD-95 triggered by NMDAR activation, the PTEN then becomes anchored to the postsynaptic membranes, driving depression of the AMPA receptor-mediated synaptic responses required for NMDAR-LTD (Jurado et al., 2010).

### FMRP

Fragile mental retardation protein (FMRP), encoded by *FMR1*, functions as an mRNA-binding translational suppressor, and is also involved in RNA-, channel- and protein binding to modulate synaptic transmission and plasticity (Davis and Broadie, 2017). Fragile X syndrome (FXS), the most common form of inherited ID and the most common monogenic cause of ASD, is caused by the loss of FMRP. 60–74% of male and 16–45% of female FXS patients meet the diagnostic criteria of ASD (Klusek et al., 2014). Nearly 1,000 high-confidence FMRP-associated transcripts have been identified (Darnell et al., 2011; Tang et al., 2015), and there is substantial overlap between putative FMRP target mRNAs and ASD-related mRNAs (Muddashetty et al., 2011; Zhang et al., 2014). Therefore, the commonly observed ASD endophenotype of FXS might reflect a “multiple hit” effect on the expression of ASD-related genes caused by loss of FMRP.

FMRP plays a key role in the dynamic regulation of the synaptic proteome. FMRP associates with *Nlgn1*, *Nlgn2* and *Nlgn3* mRNAs; consequently, *Fmr1* KO leads to elevated local translation of *Nlgn* mRNAs and increased targeting of Nlgn1 and Nlgn3 to postsynaptic membranes (Chmielewska et al., 2018). FMRP binds to a G-rich region of PSD-95 mRNA, and together with microRNA miR-125a, plays an important role in the reversible inhibition of PSD-95 mRNA translation in neurons (Muddashetty et al., 2011; Stefanovic et al., 2015). However, recently, it was revealed that FMRP enhances rather than represses the translation of large ASD-related proteins; this discrepancy may arise because many FMRP targets act negatively on translation, protein stability or cell growth (Greenblatt and Spradling, 2018).

In *Fmr1* KO mice, mGluR-LTD in the CA1 region of hippocampus is exaggerated (Hou et al., 2006; Till et al., 2015), while mGluR5 is less associated with the long Homer isoforms and more associated with short Homer 1a (Giuffrida et al., 2005; Ronesi et al., 2012). The long Homer proteins, Homer 1b, 1c, 2 and 3, target mGluR5 to the PSD though their interaction with Shank proteins, whereas the short Homer 1a uncouples mGlu5R from postsynaptic membranes (Kammermeier and Worley, 2007). Therefore, mGluR5 is significantly more mobile at hippocampal synapses of *FMR1* KO mice, causing increased co-clustering of mGluR5 and NMDAR, which correlates with reduced amplitude of NMDAR currents and the absence of NMDAR-LTD (Aloisi et al., 2017). The enhanced mGluR-LTD is likely caused by dysregulated NMDAR signaling. Blocking GluN2B rescued mGluR-LTD, suggesting that GluN2B-containing NMDARs are hyperactive in *Fmr1* KO mice (Toft et al., 2016). Moreover, NMDAR-LTP is decreased in the hippocampus of *Fmr1* KO rats and mice, and pharmacological or genetic blockage of GluN2A-containing NMDARs completely rescues LTP and mGluR-LTD (Tian et al., 2017; Lundbye et al., 2018). In addition to the mGluR hyperexcitability theory, GABAergic hypoinhibition that leads to E/I imbalance may cause disease symptoms. In mouse and *Drosophila* FXS models, the mRNA and protein levels of GABA_A_R and glutamic acid decarboxylase (GAD), the rate-limiting GABA synthesis enzyme, are downregulated (Adusei et al., 2010; Gatto et al., 2014). In the cerebellum of *Fmr1* KO mice, excessive GABA release from basket cell synapses, which results from increased excitability and Ca^2+^ transients in the presynaptic sites due to disruption of the interaction between FMRP and K_V_1.2, suppresses the firing activity of Purkinje neurons (Yang et al., 2018).

### UBE3A

Ubiquitin-protein ligase E3A (UBE3A) is encoded by *UBE3A* gene, located in an imprinted region on chromosome 15q11-q13. Loss of function of the maternally expressed *UBE3A* gene gives rise to Angelman Syndrome (AS), characterized by microcephaly, ID, motor abnormalities, profound speech impairment, epilepsy and a unique behavioral pattern, including a happy demeanor (Buiting et al., 2016). The duplication or triplication of maternally inherited 15q11-q13 is associated with ASD, accounting for 1–3% of ASD cases worldwide (Hogart et al., 2010; LaSalle et al., 2015). UBE3A transfers ubiquitin from an E2 ubiquitin-conjugating enzyme to the substrate proteins, thus tagging these substrate proteins for proteasomal degradation. Not surprisingly, function deficits of UBE3A can increase the synaptic abundance of its substrate proteins and dysregulate many synaptic processes including synapse development, elimination, and function.

*Drosophila* with *dUbe3a* mutations have excess synaptic boutons and endocytic defects at neuromuscular junction terminals due to enhanced level of bone morphogenetic protein (BMP) signaling, crucial to neurodevelopment throughout evolution (Li et al., 2016b). In *Ube3a^m-/p+^* neurons, Arc protein accumulates due to a lack of degradation, leading to the excessive internalization of AMPARs, eventually disrupting homeostatic synaptic scaling of AMPARs (Greer et al., 2010; Kuhnle et al., 2013; Pastuzyn and Shepherd, 2017). *Ube3a^m-/p+^* mice show strongly decreased inhibitory transmission and increased excitatory transmission, leading to marked E/I imbalance. This is accompanied by an abnormal accumulation of clathrin-coated vesicles at inhibitory axon terminals, suggesting a defect in vesicle cycling (Wallace et al., 2012; Judson et al., 2016; Rotaru et al., 2018). In contrast, increasing gene dosage of *Ube3a* suppresses glutamatergic, but not GABAergic, synaptic transmission as a result of reduced presynaptic release probability, synaptic glutamate concentration, and postsynaptic action potential coupling (Smith et al., 2011). Besides, excessive UBE3A dosage is found to impair retinoic acid-mediated neuronal homeostatic synaptic plasticity (Xu et al., 2018). *UBE3A^m-/p+^* mice show severely impaired hippocampal LTP despite normal neuroanatomy and basal synaptic transmission, which may be the result of increased inhibitory phosphorylation of CaMKII and enhanced neuregulin-ErbB4 signaling, so both mutations that prevent inhibitory phosphorylation of αCaMKII and ErbB inhibitors can reverse these deficits in LTP (Jiang et al., 1998; van Woerden et al., 2007; Kaphzan et al., 2012). In UBE3A-deficient mice, the level of postsynaptic SK2, a member of the family of small-conductance calcium-activated potassium channels (SKs), is elevated, resulting in decreased NMDAR activation, thereby impairing hippocampal LTP (Sun et al., 2015). mGlu5 receptor-dependent LTD is enhanced in hippocampal slices of *Ube3a ^m-/p+^* mice. However, totally unlike the coupling changes observed in FXS mice, *Ube3a ^m-/p+^* mice show a reduced expression of Homer 1a and an increased coupling of mGluR5 and Homer 1/c (Pignatelli et al., 2014).

### MeCP2

Methyl-CpG binding protein 2 (MeCP2), encoded by *MECP2* gene, is a nuclear protein that preferentially binds to methylated DNA over unmethylated DNA, leading to global transcriptional repression by recruiting corepressors, such as histone deacetylases and transcriptional silencing factors. Loss-of-function mutations in *MECP2* are the major cause of Rett Syndrome (RTT), an X-linked progressive neurodevelopmental disorder that nearly exclusively affects female patients. RTT patients experience an apparently normal postnatal development up to 6–18 months of age, and then neurological symptoms appear, including ID, autistic features, epilepsy and deteriorated motor and sensory function. Gain-of-function mutations in *MECP2* are associated with the neurodevelopmental disorder *MECP2* duplication syndrome, marked by severe ID, stunted motor development and epilepsy, as well as autistic-like features and anxiety.

MeCP2 can regulate synapse development and synaptic function, which is crucial for normal E/I balance. Hippocampal glutamatergic neurons that lack MeCP2 display reduced synaptic responses, whereas neurons with doubling of MeCP2 exhibit an enhancement in synaptic responses, primarily due to the alteration in the number of synapses formed (Chao et al., 2007). Wild-type GABAergic neurons in cerebral cortex express ∼50% more MeCP2 than non-GABAergic neurons, indicating Mecp2 might be particularly important to GABAergic function (Chao et al., 2010). MeCP2-deficient GABAergic neurons show reduced inhibitory quantal size, consistent with a presynaptic reduction of glutamic acid decarboxylase-1 (GAD-1) and GAD-2 levels (Chao et al., 2010). The expression levels of GABA transporter 1 (GAT1) and vesicular GABA transporter (vGAT) are significantly lower in the frontal cortex of *Mecp2* KO mice, implying a dysfunctional GABA recycling system (Barth et al., 2014). Loss of MeCP2 also accelerates the NMDAR subunit switch from Glu2B to 2A at excitatory synapses onto cortical PV cells, indicating an acceleration of NMDAR development, but delays this switch in pyramidal cells, suggesting a developmental delay (Mierau et al., 2016). The frequency and amplitude of mIPSCs is decreased, while the amplitude of mEPSCs is significantly enhanced in hippocampal pyramidal neurons of *Mecp2* KO male mice (*Mecp2^-/y^*) and Mecp2 A140V mutant mice (Ma et al., 2014; Calfa et al., 2015). Naïve excitatory synapses in CA1 pyramidal neurons of *Mecp2* KO mice show larger AMPAR-mediated synaptic currents that results from a higher expression of GluA1-containing receptors (Li et al., 2016a). In the somatosensory cortex of *Mecp2*-null mice, AMPAR- and NMDAR-excitation is not altered, while postsynaptic inhibition is enhanced, as demonstrated by an increase in the amplitude of GABA_A_R-mediated sIPSCs and mIPSCs (Lo et al., 2016).

LTP and LTD are both attenuated in the hippocampus of *Mecp2* KO mice (Asaka et al., 2006). The similar impairments are also observed in mice with truncated mutation of *Mecp2* (*Mecp2^308^*) and heterozygous female *Mecp2-stop* mice (*Mecp2^stop/-^*) (Moretti et al., 2006; Weng et al., 2011). However, the underlying causes of this impaired LTP and LTD have not yet been thoroughly elucidated. One plausible mechanism for impaired LTP in *Mecp2*-null mice is the lack of activity-dependent trafficking of GluA1, since long-term plasticity is critically determined by activity-dependent insertion and removal of AMPARs to and from postsynaptic membranes (Li et al., 2016a). Moreover, an excess of free regulatory protein kinase A (PKA) subunits is seen in *Mecp2^-/y^*, binding to cAMP and buffering cAMP levels to restrict its availability for PKA activation, perhaps explaining the observed defective LTP in *Mecp2^-/y^* CA1 neurons (Balakrishnan et al., 2016). Mice with MeCP2 overexpression restricted in neurons (*Tau-Mecp2*) also display impaired hippocampal LTP (Na et al., 2012). With regard to short-term plasticity, loss-of-*Mecp2* function leads to reduced paired-pulse ratios (Asaka et al., 2006; Moretti et al., 2006; Weng et al., 2011), whereas gain-of-*Mecp2* function augments paired-pulse responses (Collins et al., 2004; Na et al., 2012).

## Synaptic Elimination

Besides abnormalities in synaptogenesis, synaptic neurotrans mission, and synaptic plasticity caused by mutations in ASD-linked genes, several ASD risk genes including *MEF2C*, *FMR1*, *DLG4*, and *PCDH10* are also involved in the process of synaptic elimination, so it is appropriate to discuss them as a whole (Ram Venkataraman et al., 2017), bearing in mind that completely different genetic causes can lead to a common cellular pathway leading to synaptic defects.

Maintenance of cognition and normal behaviors is heavily dependent on precise formation of mature neuronal circuits. During postnatal brain development, synaptic formation predominates at first, resulting in synapses in excess of functional need. Then in the subsequent postnatal period, activity-dependent elimination of redundant synapses decreases the number of synapses. In general, synaptic activity and the resulting neuronal depolarization and Ca^2+^ influx activates the myocyte enhancer factor-2 (MEF2) family of transcription factors, inducing a rapid and robust synapse elimination (Wilkerson et al., 2014). *MEF2C* haploinsufficiency has been identified in patients who exhibit signs and syndromes that include ASD, ID and variable epilepsy (Novara et al., 2010; Paciorkowski et al., 2013). MEF2C acts as a convergent point in multiple regulatory pathways involved in the pathogenesis of ASD and ID (Parikshak et al., 2013; Gilissen et al., 2014).

MEF2-induced synapse elimination requires multiple autism-associated components. Deletion of MEF2C in mice causes a marked increase in the number of excitatory synapses, whereas neuronal expression of super-activating form of MEF2C leads to the reduced number of excitatory postsynaptic sites (Barbosa et al., 2008; Pfeiffer et al., 2010; Cole et al., 2012). However, MEF2 fails to eliminate functional or structural excitatory synapses in the hippocampus of *Fmr1* KO mice, demonstrating FMRP functions downstream of MEF2-induced synapse elimination, consistent with the excess dendritic spines seen in *Fmr1* KO mice and patients with FXS (Pfeiffer et al., 2010) FMRP and MEF2 cooperatively regulate the expression of protocadherin-10 (Pcdh10), a member of the cadherin superfamily of calcium-dependent cell adhesion molecules encoded by the *PCDH10* gene. MEF2 activation leads to PSD-95 ubiquitination by the ubiquitin E3 ligase Murine Double Minute-2 (Mdm2), which is then delivered by Pcdh10 to the proteasome for degradation (Tsai N. et al., 2012). Exonic CNVs in *PTEN* gene are associated with ASD (Morrow et al., 2008; Bucan et al., 2009). Mice lacking one copy of *Pcdh10 (Pcdh10^+/-^)* display reduced social approach behavior, as well as abnormal spine morphology, reduced levels of NMDAR subunits, and impaired gamma synchronization (Schoch et al., 2017). In neurons of *Fmr1* KO mice, elevating levels of eukaryotic translation elongation factor 1-alpha (EF1α), an FMRP target mRNA, prevents phosphatase 2A (PP2A) interaction with and dephosphorylation of Mdm2 upon MEF2 activation, rendering Mdm2 hyperphosphorylated and trapped in the nucleus (Tsai N. et al., 2017).

## Conclusion

Although the etiology of ASD is highly heterogeneous, numerous studies have shed light on common cellular pathways that converge at synapses. The functions of proteins encoded by ASD risk genes are so complex that in most cases, a single protein is implicated in multiple synaptic aspects, including synapse formation and elimination, synaptic transmission and plasticity. The data reviewed above suggests that the pathogenesis of ASD, at least in part, can be attributed to synaptic dysfunction. The ASD phenotype can be seen as the ultimate result of multiple hits of genetic and environmental factors. Further elucidation of the underlying synaptopathology, and advances in gene therapies and targeted drugs to modulate these processes, could lead to promising new therapies for the treatment of ASDs.

## Author Contributions

SG played a primary role in literature review and writing, revising of the manuscript. FH and CC participated literature searching. NP and LW helped with consultation of genetic information. XD and LY contributed to the qualitative coding and the writing of the manuscript. FY and JP developed the initial idea for this study, led the literature search, and critically reviewed manuscript drafts for intellectual content.

## Conflict of Interest Statement

The authors declare that the research was conducted in the absence of any commercial or financial relationships that could be construed as a potential conflict of interest.

## Footnotes

1 https://gene.sfari.org/

## References

[B1] Adusei D. C. Pacey L. K. Chen D. Hampson D. R. (2010). Early developmental alterations in GABAergic protein expression in fragile X knockout mice. *Neuropharmacology* 59 167–171. 10.1016/j.neuropharm.2010.05.002 20470805

[B2] Aloisi E. Le Corf K. Dupuis J. Zhang P. Ginger M. Labrousse V. (2017). Altered surface mGluR5 dynamics provoke synaptic NMDAR dysfunction and cognitive defects in Fmr1 knockout mice. *Nat. Commun.* 8:1103. 10.1038/s41467-017-01191-2 29062097 PMC5653653

[B3] Arons M. H. Thynne C. J. Grabrucker A. M. Li D. Schoen M. Cheyne J. E. (2012). Autism-associated mutations in ProSAP2/Shank3 impair synaptic transmission and neurexin-neuroligin-mediated transsynaptic signaling. *J. Neurosci.* 32 14966–14978. 10.1523/jneurosci.2215-12.2012 23100419 PMC3752148

[B4] Asaka Y. Jugloff D. G. Zhang L. Eubanks J. H. Fitzsimonds R. M. (2006). Hippocampal synaptic plasticity is impaired in the Mecp2-null mouse model of Rett syndrome. *Neurobiol. Dis.* 21 217–227. 10.1016/j.nbd.2005.07.005 16087343

[B5] Auerbach B. D. Osterweil E. K. Bear M. F. (2011). Mutations causing syndromic autism define an axis of synaptic pathophysiology. *Nature* 480 63–68. 10.1038/nature10658 22113615 PMC3228874

[B6] Bai Y. Qiu S. Li Y. Li Y. Zhong W. Shi M. (2018). Genetic association between SHANK2 polymorphisms and susceptibility to autism spectrum disorder. *IUBMB Life* 70 763–776. 10.1002/iub.1876 29934968

[B7] Bailey A. Le Couteur A. Gottesman I. Bolton P. Simonoff E. Yuzda E. (1995). Autism as a strongly genetic disorder: evidence from a British twin study. *Psychol. Med.* 25 63–77. 7792363 10.1017/s0033291700028099

[B8] Baio J. Wiggins L. Christensen D. L. Maenner M. J. Daniels J. Warren Z. (2018). Prevalence of autism spectrum disorder among children aged 8 years - autism and developmental disabilities monitoring network, 11 sites, United States, 2014. *MMWR Surveill. Summ.* 67 1–23. 10.15585/mmwr.ss6706a1 29701730 PMC5919599

[B9] Balakrishnan S. Niebert M. Richter D. W. (2016). Rescue of cyclic AMP mediated long term potentiation impairment in the hippocampus of Mecp2 Knockout (Mecp2(-/y) ) Mice by Rolipram. *Front. Cell Neurosci.* 10:15. 10.3389/fncel.2016.00015 26869885 PMC4737891

[B10] Barbosa A. C. Kim M. S. Ertunc M. Adachi M. Nelson E. D. McAnally J. (2008). MEF2C, a transcription factor that facilitates learning and memory by negative regulation of synapse numbers and function. *Proc. Natl. Acad. Sci. U.S.A.* 105 9391–9396. 10.1073/pnas.0802679105 18599438 PMC2453723

[B11] Barth L. Sutterlin R. Nenniger M. Vogt K. E. (2014). Reduced synaptic activity in neuronal networks derived from embryonic stem cells of murine Rett syndrome model. *Front. Cell Neurosci.* 8:79. 10.3389/fncel.2014.00079 24723848 PMC3972477

[B12] Bateup H. S. Takasaki K. T. Saulnier J. L. Denefrio C. L. Sabatini B. L. (2011). Loss of Tsc1 in vivo impairs hippocampal mGluR-LTD and increases excitatory synaptic function. *J. Neurosci.* 31 8862–8869. 10.1523/jneurosci.1617-11.2011 21677170 PMC3133739

[B13] Béïque J. Lin D. Kang M. Aizawa H. Takamiya K. Huganir R. (2006). Synapse-specific regulation of AMPA receptor function by PSD-95. *Proc. Natl. Acad. Sci. U.S.A.* 103 19535–19540. 17148601 10.1073/pnas.0608492103 PMC1748260

[B14] Berkel S. Marshall C. R. Weiss B. Howe J. Roeth R. Moog U. (2010). Mutations in the SHANK2 synaptic scaffolding gene in autism spectrum disorder and mental retardation. *Nat. Genet.* 42 489–491. 10.1038/ng.589 20473310

[B15] Biederer T. Sudhof T. C. (2000). Mints as adaptors. Direct binding to neurexins and recruitment of munc18. *J. Biol. Chem.* 275 39803–39806. 10.1074/jbc.C000656200 11036064

[B16] Blumberg S. Bramlett M. Kogan M. Schieve L. Jones J. Lu M. (2013). Changes in prevalence of parent-reported autism spectrum disorder in school-aged U.S. children: 2007 to 2011-2012. *Natl. Health Stat. Rep.* 65 1–11. 24988818

[B17] Blundell J. Blaiss C. Etherton M. Espinosa F. Tabuchi K. Walz C. (2010). Neuroligin-1 deletion results in impaired spatial memory and increased repetitive behavior. *J. Neurosci.* 30 2115–2129. 10.1523/JNEUROSCI.4517-09.2010 20147539 PMC2824441

[B18] Boccuto L. Lauri M. Sarasua S. M. Skinner C. D. Buccella D. Dwivedi A. (2013). Prevalence of SHANK3 variants in patients with different subtypes of autism spectrum disorders. *Eur. J. Hum. Genet.* 21 310–316. 10.1038/ejhg.2012.175 22892527 PMC3573207

[B19] Born G. Grayton H. M. Langhorst H. Dudanova I. Rohlmann A. Woodward B. W. (2015). Genetic targeting of NRXN2 in mice unveils role in excitatory cortical synapse function and social behaviors. *Front. Synaptic Neurosci.* 7:3. 10.3389/fnsyn.2015.00003 25745399 PMC4333794

[B20] Boukhris T. Sheehy O. Mottron L. Berard A. (2016). Antidepressant use during pregnancy and the risk of autism spectrum disorder in children. *JAMA Pediatr.* 170 117–124. 10.1001/jamapediatrics.2015.3356 26660917

[B21] Brockhaus J. Schreitmuller M. Repetto D. Klatt O. Reissner C. Elmslie K. (2018). Alpha-neurexins together with alpha2delta-1 auxiliary subunits regulate Ca(2+) influx through Cav2.1 channels. *J. Neurosci.* 38 8277–8294. 10.1523/jneurosci.0511-18.2018 30104341 PMC6596161

[B22] Brown A. S. Sourander A. Hinkka-Yli-Salomaki S. McKeague I. W. Sundvall J. Surcel H. M. (2014). Elevated maternal C-reactive protein and autism in a national birth cohort. *Mol. Psychiatry* 19 259–264. 10.1038/mp.2012.197 23337946 PMC3633612

[B23] Bucan M. Abrahams B. S. Wang K. Glessner J. T. Herman E. I. Sonnenblick L. I. (2009). Genome-wide analyses of exonic copy number variants in a family-based study point to novel autism susceptibility genes. *PLoS Genet.* 5:e1000536. 10.1371/journal.pgen.1000536 19557195 PMC2695001

[B24] Budreck E. C. Scheiffele P. (2007). Neuroligin-3 is a neuronal adhesion protein at GABAergic and glutamatergic synapses. *Eur. J. Neurosci.* 26 1738–1748. 10.1111/j.1460-9568.2007.05842.x 17897391

[B25] Buiting K. Williams C. Horsthemke B. (2016). Angelman syndrome - insights into a rare neurogenetic disorder. *Nat. Rev. Neurol.* 12 584–593. 10.1038/nrneurol.2016.133 27615419

[B26] Bustos F. J. Varela-Nallar L. Campos M. Henriquez B. Phillips M. Opazo C. (2014). PSD95 suppresses dendritic arbor development in mature hippocampal neurons by occluding the clustering of NR2B-NMDA receptors. *PLoS One* 9:e94037. 10.1371/journal.pone.0094037 24705401 PMC3976375

[B27] Butler M. G. Dasouki M. J. Zhou X. P. Talebizadeh Z. Brown M. Takahashi T. N. (2005). Subset of individuals with autism spectrum disorders and extreme macrocephaly associated with germline PTEN tumour suppressor gene mutations. *J. Med. Genet.* 42 318–321. 10.1136/jmg.2004.024646 15805158 PMC1736032

[B28] Buxbaum J. D. Cai G. Chaste P. Nygren G. Goldsmith J. Reichert J. (2007). Mutation screening of the PTEN gene in patients with autism spectrum disorders and macrocephaly. *Am. J. Med. Genet. B Neuropsychiatr. Genet.* 144b, 484–491. 10.1002/ajmg.b.30493 17427195 PMC3381648

[B29] Cajigas I. J. Will T. Schuman E. M. (2010). Protein homeostasis and synaptic plasticity. *EMBO J.* 29 2746–2752. 10.1038/emboj.2010.173 20717144 PMC2924649

[B30] Calfa G. Li W. Rutherford J. M. Pozzo-Miller L. (2015). Excitation/inhibition imbalance and impaired synaptic inhibition in hippocampal area CA3 of Mecp2 knockout mice. *Hippocampus* 25 159–168. 10.1002/hipo.22360 25209930 PMC4300269

[B31] Camacho-Garcia R. J. Planelles M. I. Margalef M. Pecero M. L. Martinez-Leal R. Aguilera F. (2012). Mutations affecting synaptic levels of neurexin-1beta in autism and mental retardation. *Neurobiol. Dis.* 47 135–143. 10.1016/j.nbd.2012.03.031 22504536

[B32] Chao H. T. Chen H. Samaco R. C. Xue M. Chahrour M. Yoo J. (2010). Dysfunction in GABA signalling mediates autism-like stereotypies and Rett syndrome phenotypes. *Nature* 468 263–269. 10.1038/nature09582 21068835 PMC3057962

[B33] Chao H. T. Zoghbi H. Y. Rosenmund C. (2007). MeCP2 controls excitatory synaptic strength by regulating glutamatergic synapse number. *Neuron* 56 58–65. 10.1016/j.neuron.2007.08.018 17920015 PMC2198899

[B34] Chen X. Nelson C. D. Li X. Winters C. A. Azzam R. Sousa A. A. (2011). PSD-95 is required to sustain the molecular organization of the postsynaptic density. *J. Neurosci.* 31 6329–6338. 10.1523/jneurosci.5968-10.2011 21525273 PMC3099547

[B35] Chih B. Afridi S. K. Clark L. Scheiffele P. (2004). Disorder-associated mutations lead to functional inactivation of neuroligins. *Hum. Mol. Genet.* 13 1471–1477. 10.1093/hmg/ddh158 15150161

[B36] Ching M. Shen Y. Tan W. Jeste S. Morrow E. Chen X. (2010). Deletions of NRXN1 (neurexin-1) predispose to a wide spectrum of developmental disorders. *Am. J. Med. Genet. B Neuropsychiatr. Genet.* 153B, 937–947. 10.1002/ajmg.b.31063 20468056 PMC3001124

[B37] Chmielewska J. J. Kuzniewska B. Milek J. Urbanska K. Dziembowska M. (2018). Neuroligin 1, 2, and 3 regulation at the synapse: FMRP-dependent translation and activity-induced proteolytic cleavage. *Mol. Neurobiol.* 10.1007/s12035-018-1243-1 [Epub ahead of print]. 30056576 PMC6459971

[B38] Chubykin A. Atasoy D. Etherton M. Brose N. Kavalali E. Gibson J. (2007). Activity-dependent validation of excitatory versus inhibitory synapses by neuroligin-1 versus neuroligin-2. *Neuron* 54 919–931. 17582332 10.1016/j.neuron.2007.05.029 PMC3738748

[B39] Cole C. J. Mercaldo V. Restivo L. Yiu A. P. Sekeres M. J. Han J. H. (2012). MEF2 negatively regulates learning-induced structural plasticity and memory formation. *Nat. Neurosci.* 15 1255–1264. 10.1038/nn.3189 22885849

[B40] Collins A. L. Levenson J. M. Vilaythong A. P. Richman R. Armstrong D. L. Noebels J. L. (2004). Mild overexpression of MeCP2 causes a progressive neurological disorder in mice. *Hum. Mol. Genet.* 13 2679–2689. 10.1093/hmg/ddh282 15351775

[B41] Colvert E. Tick B. McEwen F. Stewart C. Curran S. R. Woodhouse E. (2015). Heritability of autism spectrum disorder in a UK population-based twin sample. *JAMA Psychiatry* 72 415–423. 10.1001/jamapsychiatry.2014.3028 25738232 PMC4724890

[B42] Comoletti D. De Jaco A. Jennings L. L. Flynn R. E. Gaietta G. Tsigelny I. (2004). The Arg451Cys-neuroligin-3 mutation associated with autism reveals a defect in protein processing. *J. Neurosci.* 24 4889–4893. 10.1523/jneurosci.0468-04.2004 15152050 PMC6729460

[B43] Costa V. Aigner S. Vukcevic M. Sauter E. Behr K. Ebeling M. (2016). mTORC1 inhibition corrects neurodevelopmental and synaptic alterations in a human stem cell model of tuberous sclerosis. *Cell Rep.* 15 86–95. 10.1016/j.celrep.2016.02.090 27052171

[B44] Cupolillo D. Hoxha E. Faralli A. De Luca A. Rossi F. Tempia F. (2016). Autistic-like traits and cerebellar dysfunction in purkinje Cell PTEN knock-out mice. *Neuropsychopharmacology* 41 1457–1466. 10.1038/npp.2015.339 26538449 PMC4832032

[B45] Dachtler J. Glasper J. Cohen R. N. Ivorra J. L. Swiffen D. J. Jackson A. J. (2014). Deletion of alpha-neurexin II results in autism-related behaviors in mice. *Transl. Psychiatry* 4:e484. 10.1038/tp.2014.123 25423136 PMC4259993

[B46] Dachtler J. Ivorra J. Rowland T. Lever C. Rodgers R. Clapcote S. (2015). Heterozygous deletion of α-neurexin I or α-neurexin II results in behaviors relevant to autism and schizophrenia. *Behav. Neurosci.* 129 765–776. 10.1037/bne0000108 26595880 PMC4655861

[B47] Dahlhaus R. Hines R. Eadie B. Kannangara T. Hines D. Brown C. (2010). Overexpression of the cell adhesion protein neuroligin-1 induces learning deficits and impairs synaptic plasticity by altering the ratio of excitation to inhibition in the hippocampus. *Hippocampus* 20 305–322. 10.1002/hipo.20630 19437420

[B48] Darnell J. C. Van Driesche S. J. Zhang C. Hung K. Y. Mele A. Fraser C. E. (2011). FMRP stalls ribosomal translocation on mRNAs linked to synaptic function and autism. *Cell* 146 247–261. 10.1016/j.cell.2011.06.013 21784246 PMC3232425

[B49] Davis J. K. Broadie K. (2017). Multifarious functions of the fragile X mental retardation protein. *Trends Genet.* 33 703–714. 10.1016/j.tig.2017.07.008 28826631 PMC5610095

[B50] Dejanovic B. Djemie T. Grunewald N. Suls A. Kress V. Hetsch F. (2015). Simultaneous impairment of neuronal and metabolic function of mutated gephyrin in a patient with epileptic encephalopathy. *EMBO Mol. Med.* 7 1580–1594. 10.15252/emmm.201505323 26613940 PMC4693503

[B51] Dejanovic B. Lal D. Catarino C. B. Arjune S. Belaidi A. A. Trucks H. (2014a). Exonic microdeletions of the gephyrin gene impair GABAergic synaptic inhibition in patients with idiopathic generalized epilepsy. *Neurobiol. Dis.* 67 88–96. 10.1016/j.nbd.2014.02.001 24561070

[B52] Dejanovic B. Schwarz G. (2014). Neuronal nitric oxide synthase-dependent S-nitrosylation of gephyrin regulates gephyrin clustering at GABAergic synapses. *J. Neurosci.* 34 7763–7768. 10.1523/jneurosci.0531-14.2014 24899700 PMC6608267

[B53] Dejanovic B. Semtner M. Ebert S. Lamkemeyer T. Neuser F. Luscher B. (2014b). Palmitoylation of gephyrin controls receptor clustering and plasticity of GABAergic synapses. *PLoS Biol.* 12:e1001908. 10.1371/journal.pbio.1001908 25025157 PMC4099074

[B54] Doshi-Velez F. Ge Y. Kohane I. (2014). Comorbidity clusters in autism spectrum disorders: an electronic health record time-series analysis. *Pediatrics* 133 e54–e63. 10.1542/peds.2013-0819 24323995 PMC3876178

[B55] Durand C. M. Betancur C. Boeckers T. M. Bockmann J. Chaste P. Fauchereau F. (2007). Mutations in the gene encoding the synaptic scaffolding protein SHANK3 are associated with autism spectrum disorders. *Nat. Genet.* 39 25–27. 10.1038/ng1933 17173049 PMC2082049

[B56] Egger G. Roetzer K. M. Noor A. Lionel A. C. Mahmood H. Schwarzbraun T. (2014). Identification of risk genes for autism spectrum disorder through copy number variation analysis in Austrian families. *Neurogenetics* 15 117–127. 10.1007/s10048-014-0394-0 24643514

[B57] Ehninger D. Han S. Shilyansky C. Zhou Y. Li W. Kwiatkowski D. J. (2008). Reversal of learning deficits in a Tsc2+/- mouse model of tuberous sclerosis. *Nat. Med.* 14 843–848. 10.1038/nm1788 18568033 PMC2664098

[B58] Estes M. L. McAllister A. K. (2016). Maternal immune activation: implications for neuropsychiatric disorders. *Science* 353 772–777. 10.1126/science.aag3194 27540164 PMC5650490

[B59] Etherton M. Blaiss C. Powell C. Südhof T. (2009). Mouse neurexin-1alpha deletion causes correlated electrophysiological and behavioral changes consistent with cognitive impairments. *Proc. Natl. Acad. Sci. U.S.A.* 106 17998–18003. 10.1073/pnas.0910297106 19822762 PMC2764944

[B60] Etherton M. Földy C. Sharma M. Tabuchi K. Liu X. Shamloo M. (2011a). Autism-linked neuroligin-3 R451C mutation differentially alters hippocampal and cortical synaptic function. *Proc. Natl. Acad. Sci. U.S.A.* 108 13764–13769. 10.1073/pnas.1111093108 21808020 PMC3158170

[B61] Etherton M. Tabuchi K. Sharma M. Ko J. Südhof T. (2011b). An autism-associated point mutation in the neuroligin cytoplasmic tail selectively impairs AMPA receptor-mediated synaptic transmission in hippocampus. *EMBO J.* 30 2908–2919. 10.1038/emboj.2011.182 21642956 PMC3160244

[B62] Feng W. Zhang M. (2009). Organization and dynamics of PDZ-domain-related supramodules in the postsynaptic density. *Nat. Rev. Neurosci.* 10 87–99. 10.1038/nrn2540 19153575

[B63] Flores C. E. Nikonenko I. Mendez P. Fritschy J. M. Tyagarajan S. K. Muller D. (2015). Activity-dependent inhibitory synapse remodeling through gephyrin phosphorylation. *Proc. Natl. Acad. Sci. U.S.A.* 112 E65–E72. 10.1073/pnas.1411170112 25535349 PMC4291629

[B64] Folstein S. Rutter M. (1977). Infantile autism: a genetic study of 21 twin pairs. *J. Child Psychol. Psychiatry* 18 297–321. 562353 10.1111/j.1469-7610.1977.tb00443.x

[B65] Gardener H. Spiegelman D. Buka S. L. (2009). Prenatal risk factors for autism: comprehensive meta-analysis. *Br. J. Psychiatry* 195 7–14. 10.1192/bjp.bp.108.051672 19567888 PMC3712619

[B66] Gatto C. L. Broadie K. (2010). Genetic controls balancing excitatory and inhibitory synaptogenesis in neurodevelopmental disorder models. *Front. Synaptic Neurosci.* 2:4. 10.3389/fnsyn.2010.00004 21423490 PMC3059704

[B67] Gatto C. L. Pereira D. Broadie K. (2014). GABAergic circuit dysfunction in the Drosophila Fragile X syndrome model. *Neurobiol. Dis.* 65 142–159. 10.1016/j.nbd.2014.01.008 24423648 PMC3988906

[B68] Gaugler T. Klei L. Sanders S. J. Bodea C. A. Goldberg A. P. Lee A. B. (2014). Most genetic risk for autism resides with common variation. *Nat. Genet.* 46 881–885. 10.1038/ng.3039 25038753 PMC4137411

[B69] Gauthier J. Siddiqui T. J. Huashan P. Yokomaku D. Hamdan F. F. Champagne N. (2011). Truncating mutations in NRXN2 and NRXN1 in autism spectrum disorders and schizophrenia. *Hum. Genet.* 130 563–573. 10.1007/s00439-011-0975-z 21424692 PMC3204930

[B70] Gauthier J. Spiegelman D. Piton A. Lafreniere R. G. Laurent S. St-Onge J. (2009). Novel de novo SHANK3 mutation in autistic patients. *Am. J. Med. Genet. B Neuropsychiatr. Genet.* 150b, 421–424. 10.1002/ajmg.b.30822 18615476

[B71] Ghosh H. Auguadri L. Battaglia S. Simone Thirouin Z. Zemoura K. Messner S. (2016). Several posttranslational modifications act in concert to regulate gephyrin scaffolding and GABAergic transmission. *Nat. Commun.* 7:13365. 10.1038/ncomms13365 27819299 PMC5103071

[B72] Gilbert J. Man H. Y. (2017). Fundamental elements in autism: from neurogenesis and neurite growth to synaptic plasticity. *Front. Cell Neurosci.* 11:359. 10.3389/fncel.2017.00359 29209173 PMC5701944

[B73] Gilissen C. Hehir-Kwa J. Y. Thung D. T. van de Vorst M. van Bon B. W. Willemsen M. H. (2014). Genome sequencing identifies major causes of severe intellectual disability. *Nature* 511 344–347. 10.1038/nature13394 24896178

[B74] Giuffrida R. Musumeci S. D’Antoni S. Bonaccorso C. M. Giuffrida-Stella A. M. Oostra B. A. (2005). A reduced number of metabotropic glutamate subtype 5 receptors are associated with constitutive homer proteins in a mouse model of fragile X syndrome. *J. Neurosci.* 25 8908–8916. 10.1523/jneurosci.0932-05.2005 16192381 PMC6725593

[B75] Glessner J. T. Wang K. Cai G. Korvatska O. Kim C. E. Wood S. (2009). Autism genome-wide copy number variation reveals ubiquitin and neuronal genes. *Nature* 459 569–573. 10.1038/nature07953 19404257 PMC2925224

[B76] Greenblatt E. J. Spradling A. C. (2018). Fragile X mental retardation 1 gene enhances the translation of large autism-related proteins. *Science* 361 709–712. 10.1126/science.aas9963 30115809 PMC6905618

[B77] Greer P. L. Hanayama R. Bloodgood B. L. Mardinly A. R. Lipton D. M. Flavell S. W. (2010). The Angelman Syndrome protein Ube3A regulates synapse development by ubiquitinating arc. *Cell* 140 704–716. 10.1016/j.cell.2010.01.026 20211139 PMC2843143

[B78] Groeneweg F. L. Trattnig C. Kuhse J. Nawrotzki R. A. Kirsch J. (2018). Gephyrin: a key regulatory protein of inhibitory synapses and beyond. *Histochem. Cell Biol.* 150 489–508. 10.1007/s00418-018-1725-2 30264265

[B79] Guo H. Peng Y. Hu Z. Li Y. Xun G. Ou J. (2017). Genome-wide copy number variation analysis in a Chinese autism spectrum disorder cohort. *Sci. Rep.* 7:44155. 10.1038/srep44155 28281572 PMC5345089

[B80] Hackett A. Tarpey P. S. Licata A. Cox J. Whibley A. Boyle J. (2010). CASK mutations are frequent in males and cause X-linked nystagmus and variable XLMR phenotypes. *Eur. J. Hum. Genet.* 18 544–552. 10.1038/ejhg.2009.220 20029458 PMC2987321

[B81] Hammer M. Krueger-Burg D. Tuffy L. P. Cooper B. H. Taschenberger H. Goswami S. P. (2015). Perturbed hippocampal synaptic inhibition and gamma-oscillations in a Neuroligin-4 knockout mouse model of autism. *Cell Rep.* 13 516–523. 10.1016/j.celrep.2015.09.011 26456829 PMC5862414

[B82] Harris K. P. Akbergenova Y. Cho R. W. Baas-Thomas M. S. Littleton J. T. (2016). Shank modulates postsynaptic wnt signaling to regulate synaptic development. *J. Neurosci.* 36 5820–5832. 10.1523/jneurosci.4279-15.2016 27225771 PMC4879199

[B83] Hippler K. Klicpera C. (2003). A retrospective analysis of the clinical case records of ’autistic psychopaths’ diagnosed by Hans Asperger and his team at the University Children’s Hospital, Vienna. *Philos. Trans. R. Soc. Lond. B Biol. Sci.* 358 291–301. 10.1098/rstb.2002.1197 12639327 PMC1693115

[B84] Hobert J. A. Embacher R. Mester J. L. Frazier T. W. II Eng C. (2014). Biochemical screening and PTEN mutation analysis in individuals with autism spectrum disorders and macrocephaly. *Eur. J. Hum. Genet.* 22 273–276. 10.1038/ejhg.2013.114 23695273 PMC3895634

[B85] Hogart A. Wu D. LaSalle J. Schanen N. (2010). The comorbidity of autism with the genomic disorders of chromosome 15q11.2-q13. *Neurobiol. Dis.* 38 181–191. 10.1016/j.nbd.2008.08.011 18840528 PMC2884398

[B86] Hou L. Antion M. D. Hu D. Spencer C. M. Paylor R. Klann E. (2006). Dynamic translational and proteasomal regulation of fragile X mental retardation protein controls mGluR-dependent long-term depression. *Neuron* 51 441–454. 10.1016/j.neuron.2006.07.005 16908410

[B87] Hu J. Sathanoori M. Kochmar S. Azage M. Mann S. Madan-Khetarpal S. (2015). A novel maternally inherited 8q24.3 and a rare paternally inherited 14q23.3 CNVs in a family with neurodevelopmental disorders. *Am. J. Med. Genet. A* 167a, 1921–1926. 10.1002/ajmg.a.37110 25866352

[B88] Huang X. Stodieck S. K. Goetze B. Cui L. Wong M. H. Wenzel C. (2015). Progressive maturation of silent synapses governs the duration of a critical period. *Proc. Natl. Acad. Sci. U.S.A.* 112 E3131–E3140. 10.1073/pnas.1506488112 26015564 PMC4475980

[B89] Huguet G. Ey E. Bourgeron T. (2013). The genetic landscapes of autism spectrum disorders. *Annu. Rev. Genomics Hum. Genet.* 14 191–213. 10.1146/annurev-genom-091212-153431 23875794

[B90] Hultman C. M. Sandin S. Levine S. Z. Lichtenstein P. Reichenberg A. (2011). Advancing paternal age and risk of autism: new evidence from a population-based study and a meta-analysis of epidemiological studies. *Mol. Psychiatry* 16 1203–1212. 10.1038/mp.2010.121 21116277

[B91] Hung A. Y. Futai K. Sala C. Valtschanoff J. G. Ryu J. Woodworth M. A. (2008). Smaller dendritic spines, weaker synaptic transmission, but enhanced spatial learning in mice lacking Shank1. *J. Neurosci.* 28 1697–1708. 10.1523/jneurosci.3032-07.2008 18272690 PMC2633411

[B92] Huttenlocher P. R. Dabholkar A. S. (1997). Regional differences in synaptogenesis in human cerebral cortex. *J. Comp. Neurol.* 387 167–178. 9336221 10.1002/(sici)1096-9861(19971020)387:2<167::aid-cne1>3.0.co;2-z

[B93] Jaramillo T. Liu S. Pettersen A. Birnbaum S. Powell C. (2014). Autism-related neuroligin-3 mutation alters social behavior and spatial learning. *Autism Res.* 7 264–272. 10.1002/aur.1362 24619977 PMC3989414

[B94] Jiang Y. H. Armstrong D. Albrecht U. Atkins C. M. Noebels J. L. Eichele G. (1998). Mutation of the Angelman ubiquitin ligase in mice causes increased cytoplasmic p53 and deficits of contextual learning and long-term potentiation. *Neuron* 21 799–811. 9808466 10.1016/s0896-6273(00)80596-6

[B95] Judson M. C. Wallace M. L. Sidorov M. S. Burette A. C. Gu B. van Woerden G. M. (2016). GABAergic neuron-specific loss of Ube3a causes angelman syndrome-like EEG abnormalities and enhances seizure susceptibility. *Neuron* 90 56–69. 10.1016/j.neuron.2016.02.040 27021170 PMC4824651

[B96] Jurado S. Benoist M. Lario A. Knafo S. Petrok C. N. Esteban J. A. (2010). PTEN is recruited to the postsynaptic terminal for NMDA receptor-dependent long-term depression. *EMBO J.* 29 2827–2840. 10.1038/emboj.2010.160 20628354 PMC2924645

[B97] Kammermeier P. J. Worley P. F. (2007). Homer 1a uncouples metabotropic glutamate receptor 5 from postsynaptic effectors. *Proc. Natl. Acad. Sci. U.S.A.* 104 6055–6060. 10.1073/pnas.0608991104 17389377 PMC1851615

[B98] Kanner L. (1995). [Follow-up study of eleven autistic children originally reported in 1943. 1971]. *Psychiatr. Enfant* 38 421–461. 8657796

[B99] Kaphzan H. Hernandez P. Jung J. I. Cowansage K. K. Deinhardt K. Chao M. V. (2012). Reversal of impaired hippocampal long-term potentiation and contextual fear memory deficits in Angelman syndrome model mice by ErbB inhibitors. *Biol. Psychiatry* 72 182–190. 10.1016/j.biopsych.2012.01.021 22381732 PMC3368039

[B100] Kelly E. Schaeffer S. M. Dhamne S. C. Lipton J. O. Lindemann L. Honer M. (2018). mGluR5 modulation of behavioral and epileptic phenotypes in a mouse model of tuberous sclerosis complex. *Neuropsychopharmacology* 43 1457–1465. 10.1038/npp.2017.295 29206810 PMC5916364

[B101] Kim Y. S. Leventhal B. L. Koh Y. J. Fombonne E. Laska E. Lim E. C. (2011). Prevalence of autism spectrum disorders in a total population sample. *Am. J. Psychiatry* 168 904–912. 10.1176/appi.ajp.2011.10101532 21558103

[B102] Klein M. E. Monday H. Jordan B. A. (2016). Proteostasis and RNA binding proteins in synaptic plasticity and in the pathogenesis of neuropsychiatric disorders. *Neural Plast.* 2016:3857934. 10.1155/2016/3857934 26904297 PMC4745388

[B103] Klusek J. Martin G. E. Losh M. (2014). Consistency between research and clinical diagnoses of autism among boys and girls with fragile X syndrome. *J. Intellect. Disabil. Res.* 58 940–952. 10.1111/jir.12121 24528851 PMC4207708

[B104] Krueger D. D. Tuffy L. P. Papadopoulos T. Brose N. (2012). The role of neurexins and neuroligins in the formation, maturation, and function of vertebrate synapses. *Curr. Opin. Neurobiol.* 22 412–422. 10.1016/j.conb.2012.02.012 22424845

[B105] Kuhnle S. Mothes B. Matentzoglu K. Scheffner M. (2013). Role of the ubiquitin ligase E6AP/UBE3A in controlling levels of the synaptic protein Arc. *Proc. Natl. Acad. Sci. U.S.A.* 110 8888–8893. 10.1073/pnas.1302792110 23671107 PMC3670309

[B106] LaSalle J. M. Reiter L. T. Chamberlain S. J. (2015). Epigenetic regulation of UBE3A and roles in human neurodevelopmental disorders. *Epigenomics* 7 1213–1228. 10.2217/epi.15.70 26585570 PMC4709177

[B107] Lavelle T. A. Weinstein M. C. Newhouse J. P. Munir K. Kuhlthau K. A. Prosser L. A. (2014). Economic burden of childhood autism spectrum disorders. *Pediatrics* 133 e520–e529. 10.1542/peds.2013-0763 24515505 PMC7034397

[B108] Leblond C. S. Nava C. Polge A. Gauthier J. Huguet G. Lumbroso S. (2014). Meta-analysis of SHANK Mutations in Autism Spectrum Disorders: a gradient of severity in cognitive impairments. *PLoS Genet.* 10:e1004580. 10.1371/journal.pgen.1004580 25188300 PMC4154644

[B109] Lee S. Lee E. Kim R. Kim J. Lee S. Park H. (2018). Shank2 deletion in parvalbumin neurons leads to moderate hyperactivity, enhanced self-grooming and suppressed seizure susceptibility in mice. *Front. Mol. Neurosci.* 11:209. 10.3389/fnmol.2018.00209 29970987 PMC6018407

[B110] Leigh J. P. Du J. (2015). Brief report: forecasting the economic burden of autism in 2015 and 2025 in the United States. *J. Autism. Dev. Disord.* 45 4135–4139. 10.1007/s10803-015-2521-7 26183723

[B111] Letellier M. Sziber Z. Chamma I. Saphy C. Papasideri I. Tessier B. (2018). A unique intracellular tyrosine in neuroligin-1 regulates AMPA receptor recruitment during synapse differentiation and potentiation. *Nat. Commun.* 9:3979. 10.1038/s41467-018-06220-2 30266896 PMC6162332

[B112] Levinson J. N. Chery N. Huang K. Wong T. P. Gerrow K. Kang R. (2005). Neuroligins mediate excitatory and inhibitory synapse formation: involvement of PSD-95 and neurexin-1beta in neuroligin-induced synaptic specificity. *J. Biol. Chem.* 280 17312–17319. 10.1074/jbc.M413812200 15723836

[B113] Li W. Xu X. Pozzo-Miller L. (2016a). Excitatory synapses are stronger in the hippocampus of Rett syndrome mice due to altered synaptic trafficking of AMPA-type glutamate receptors. *Proc. Natl. Acad. Sci. U.S.A.* 113 E1575–E1584. 10.1073/pnas.1517244113 26929363 PMC4801299

[B114] Li W. Yao A. Zhi H. Kaur K. Zhu Y. C. Jia M. (2016b). Angelman syndrome protein Ube3a regulates synaptic growth and endocytosis by inhibiting BMP signaling in Drosophila. *PLoS Genet.* 12:e1006062. 10.1371/journal.pgen.1006062 27232889 PMC4883773

[B115] Lim C. S. Kim H. Yu N. K. Kang S. J. Kim T. Ko H. G. (2017). Enhancing inhibitory synaptic function reverses spatial memory deficits in Shank2 mutant mice. *Neuropharmacology* 112(Pt A), 104–112. 10.1016/j.neuropharm.2016.08.016 27544825

[B116] Lionel A. C. Vaags A. K. Sato D. Gazzellone M. J. Mitchell E. B. Chen H. Y. (2013). Rare exonic deletions implicate the synaptic organizer Gephyrin (GPHN) in risk for autism, schizophrenia and seizures. *Hum. Mol. Genet.* 22 2055–2066. 10.1093/hmg/ddt056 23393157

[B117] Lo F. S. Blue M. E. Erzurumlu R. S. (2016). Enhancement of postsynaptic GABAA and extrasynaptic NMDA receptor-mediated responses in the barrel cortex of Mecp2-null mice. *J. Neurophysiol.* 115 1298–1306. 10.1152/jn.00944.2015 26683074 PMC4808090

[B118] Louros S. R. Osterweil E. K. (2016). Perturbed proteostasis in autism spectrum disorders. *J. Neurochem.* 139 1081–1092. 10.1111/jnc.13723 27365114 PMC5215415

[B119] Luikart B. W. Schnell E. Washburn E. K. Bensen A. L. Tovar K. R. Westbrook G. L. (2011). Pten knockdown in vivo increases excitatory drive onto dentate granule cells. *J. Neurosci.* 31 4345–4354. 10.1523/jneurosci.0061-11.2011 21411674 PMC3113533

[B120] Lundbye C. J. Toft A. K. H. Banke T. G. (2018). Inhibition of GluN2A NMDA receptors ameliorates synaptic plasticity deficits in the Fmr1(-/y) mouse model. *J. Physiol.* 596 5017–5031. 10.1113/jp276304 30132892 PMC6187029

[B121] Ma L. Y. Wu C. Jin Y. Gao M. Li G. H. Turner D. (2014). Electrophysiological phenotypes of MeCP2 A140V mutant mouse model. *CNS Neurosci. Ther.* 20 420–428. 10.1111/cns.12229 24750778 PMC6493136

[B122] Mao W. Watanabe T. Cho S. Frost J. L. Truong T. Zhao X. (2015). Shank1 regulates excitatory synaptic transmission in mouse hippocampal parvalbumin-expressing inhibitory interneurons. *Eur. J. Neurosci.* 41 1025–1035. 10.1111/ejn.12877 25816842 PMC4405481

[B123] Marshall C. R. Noor A. Vincent J. B. Lionel A. C. Feuk L. Skaug J. (2008). Structural variation of chromosomes in autism spectrum disorder. *Am. J. Hum. Genet.* 82 477–488. 10.1016/j.ajhg.2007.12.009 18252227 PMC2426913

[B124] McBride K. L. Varga E. A. Pastore M. T. Prior T. W. Manickam K. Atkin J. F. (2010). Confirmation study of PTEN mutations among individuals with autism or developmental delays/mental retardation and macrocephaly. *Autism Res.* 3 137–141. 10.1002/aur.132 20533527

[B125] Meador K. J. Baker G. A. Browning N. Cohen M. J. Bromley R. L. Clayton-Smith J. (2013). Fetal antiepileptic drug exposure and cognitive outcomes at age 6 years (NEAD study): a prospective observational study. *Lancet Neurol.* 12 244–252. 10.1016/s1474-4422(12)70323-x 23352199 PMC3684942

[B126] Mierau S. B. Patrizi A. Hensch T. K. Fagiolini M. (2016). Cell-specific regulation of N-Methyl-D-Aspartate receptor maturation by Mecp2 in Cortical Circuits. *Biol. Psychiatry* 79 746–754. 10.1016/j.biopsych.2015.05.018 26185009 PMC4670611

[B127] Mills J. D. Iyer A. M. van Scheppingen J. Bongaarts A. Anink J. J. Janssen B. (2017). Coding and small non-coding transcriptional landscape of tuberous sclerosis complex cortical tubers: implications for pathophysiology and treatment. *Sci. Rep.* 7:8089. 10.1038/s41598-017-06145-8 28808237 PMC5556011

[B128] Mitchell R. Barton S. Harvey A. S. Williams K. (2017). Risk factors for the development of autism spectrum disorder in children with tuberous sclerosis complex: protocol for a systematic review. *Syst. Rev.* 6:49. 10.1186/s13643-017-0448-0 28270230 PMC5341363

[B129] Monaco S. A. Gulchina Y. Gao W. J. (2015). NR2B subunit in the prefrontal cortex: a double-edged sword for working memory function and psychiatric disorders. *Neurosci. Biobehav. Rev.* 56 127–138. 10.1016/j.neubiorev.2015.06.022 26143512 PMC4567400

[B130] Monteiro P. Feng G. (2017). SHANK proteins: roles at the synapse and in autism spectrum disorder. *Nat. Rev. Neurosci.* 18 147–157. 10.1038/nrn.2016.183 28179641

[B131] Moretti P. Levenson J. M. Battaglia F. Atkinson R. Teague R. Antalffy B. (2006). Learning and memory and synaptic plasticity are impaired in a mouse model of Rett syndrome. *J. Neurosci.* 26 319–327. 10.1523/jneurosci.2623-05.2006 16399702 PMC6674314

[B132] Morrow E. M. Yoo S. Y. Flavell S. W. Kim T. K. Lin Y. Hill R. S. (2008). Identifying autism loci and genes by tracing recent shared ancestry. *Science* 321 218–223. 10.1126/science.1157657 18621663 PMC2586171

[B133] Moutton S. Bruel A. L. Assoum M. Chevarin M. Sarrazin E. Goizet C. (2018). Truncating variants of the DLG4 gene are responsible for intellectual disability with marfanoid features. *Clin. Genet.* 93 1172–1178. 10.1111/cge.13243 29460436

[B134] Muddashetty R. S. Nalavadi V. C. Gross C. Yao X. Xing L. Laur O. (2011). Reversible inhibition of PSD-95 mRNA translation by miR-125a, FMRP phosphorylation, and mGluR signaling. *Mol. Cell.* 42 673–688. 10.1016/j.molcel.2011.05.006 21658607 PMC3115785

[B135] Na E. S. Nelson E. D. Adachi M. Autry A. E. Mahgoub M. A. Kavalali E. T. (2012). A mouse model for MeCP2 duplication syndrome: MeCP2 overexpression impairs learning and memory and synaptic transmission. *J. Neurosci.* 32 3109–3117. 10.1523/jneurosci.6000-11.2012 22378884 PMC3835557

[B136] Najm J. Horn D. Wimplinger I. Golden J. A. Chizhikov V. V. Sudi J. (2008). Mutations of CASK cause an X-linked brain malformation phenotype with microcephaly and hypoplasia of the brainstem and cerebellum. *Nat. Genet.* 40 1065–1067. 10.1038/ng.194 19165920

[B137] Nelson S. B. Valakh V. (2015). Excitatory/inhibitory balance and circuit homeostasis in autism spectrum disorders. *Neuron* 87 684–698. 10.1016/j.neuron.2015.07.033 26291155 PMC4567857

[B138] Novara F. Beri S. Giorda R. Ortibus E. Nageshappa S. Darra F. (2010). Refining the phenotype associated with MEF2C haploinsufficiency. *Clin. Genet.* 78 471–477. 10.1111/j.1399-0004.2010.01413.x 20412115

[B139] Orrico A. Galli L. Buoni S. Orsi A. Vonella G. Sorrentino V. (2009). Novel PTEN mutations in neurodevelopmental disorders and macrocephaly. *Clin. Genet.* 75 195–198. 10.1111/j.1399-0004.2008.01074.x 18759867

[B140] Paciorkowski A. R. Traylor R. N. Rosenfeld J. A. Hoover J. M. Harris C. J. Winter S. (2013). MEF2C Haploinsufficiency features consistent hyperkinesis, variable epilepsy, and has a role in dorsal and ventral neuronal developmental pathways. *Neurogenetics* 14 99–111. 10.1007/s10048-013-0356-y 23389741 PMC3773516

[B141] Pak C. Danko T. Zhang Y. Aoto J. Anderson G. Maxeiner S. (2015). Human neuropsychiatric disease modeling using conditional deletion reveals synaptic transmission defects caused by heterozygous mutations in NRXN1. *Cell Stem Cell* 17 316–328. 10.1016/j.stem.2015.07.017 26279266 PMC4560990

[B142] Parikshak N. N. Luo R. Zhang A. Won H. Lowe J. K. Chandran V. (2013). Integrative functional genomic analyses implicate specific molecular pathways and circuits in autism. *Cell* 155 1008–1021. 10.1016/j.cell.2013.10.031 24267887 PMC3934107

[B143] Pastuzyn E. D. Shepherd J. D. (2017). Activity-dependent arc expression and homeostatic synaptic plasticity are altered in neurons from a mouse model of angelman syndrome. *Front. Mol. Neurosci.* 10:234. 10.3389/fnmol.2017.00234 28804447 PMC5532393

[B144] Peca J. Feliciano C. Ting J. T. Wang W. Wells M. F. Venkatraman T. N. (2011). Shank3 mutant mice display autistic-like behaviours and striatal dysfunction. *Nature* 472 437–442. 10.1038/nature09965 21423165 PMC3090611

[B145] Petrini E. M. Ravasenga T. Hausrat T. J. Iurilli G. Olcese U. Racine V. (2014). Synaptic recruitment of gephyrin regulates surface GABAA receptor dynamics for the expression of inhibitory LTP. *Nat. Commun.* 5:3921. 10.1038/ncomms4921 24894704 PMC4059940

[B146] Pfeiffer B. E. Zang T. Wilkerson J. R. Taniguchi M. Maksimova M. A. Smith L. N. (2010). Fragile X mental retardation protein is required for synapse elimination by the activity-dependent transcription factor MEF2. *Neuron* 66 191–197. 10.1016/j.neuron.2010.03.017 20434996 PMC2864778

[B147] Phelan K. McDermid H. E. (2012). The 22q13.3 Deletion Syndrome (Phelan-McDermid Syndrome). *Mol. Syndromol.* 2 186–201. 22670140 10.1159/000334260 PMC3366702

[B148] Phelan M. C. Rogers R. C. Saul R. A. Stapleton G. A. Sweet K. McDermid H. (2001). 22q13 deletion syndrome. *Am. J. Med. Genet.* 101 91–99. 11391650 10.1002/1096-8628(20010615)101:2<91::aid-ajmg1340>3.0.co;2-c

[B149] Pignatelli M. Piccinin S. Molinaro G. Di Menna L. Riozzi B. Cannella M. (2014). Changes in mGlu5 receptor-dependent synaptic plasticity and coupling to homer proteins in the hippocampus of Ube3A hemizygous mice modeling angelman syndrome. *J. Neurosci.* 34 4558–4566. 10.1523/jneurosci.1846-13.2014 24672001 PMC6608124

[B150] Prasad A. Merico D. Thiruvahindrapuram B. Wei J. Lionel A. C. Sato D. (2012). A discovery resource of rare copy number variations in individuals with autism spectrum disorder. *G3* 2 1665–1685. 10.1534/g3.112.004689 23275889 PMC3516488

[B151] Ram Venkataraman G. O’Connell C. Egawa F. Kashef-Haghighi D. Wall D. P. (2017). De novo mutations in autism implicate the synaptic elimination network. *Pac. Symp. Biocomput.* 22 521–532. 10.1142/9789813207813_0048 27897003

[B152] Rasalam A. D. Hailey H. Williams J. H. Moore S. J. Turnpenny P. D. Lloyd D. J. (2005). Characteristics of fetal anticonvulsant syndrome associated autistic disorder. *Dev. Med. Child Neurol.* 47 551–555. 16108456 10.1017/s0012162205001076

[B153] Reissner C. Runkel F. Missler M. (2013). Neurexins. *Genome Biol.* 14:213. 10.1186/gb-2013-14-9-213 24083347 PMC4056431

[B154] Richards C. Jones C. Groves L. Moss J. Oliver C. (2015). Prevalence of autism spectrum disorder phenomenology in genetic disorders: a systematic review and meta-analysis. *Lancet Psychiatry* 2 909–916. 10.1016/s2215-0366(15)00376-4 26341300

[B155] Rinaldi T. Kulangara K. Antoniello K. Markram H. (2007). Elevated NMDA receptor levels and enhanced postsynaptic long-term potentiation induced by prenatal exposure to valproic acid. *Proc. Natl. Acad. Sci. U.S.A.* 104 13501–13506. 10.1073/pnas.0704391104 17675408 PMC1948920

[B156] Ritvo E. R. Freeman B. J. Mason-Brothers A. Mo A. Ritvo A. M. (1985). Concordance for the syndrome of autism in 40 pairs of afflicted twins. *Am. J. Psychiatry* 142 74–77. 10.1176/ajp.142.1.74 4038442

[B157] Rizo J. Xu J. (2015). The synaptic vesicle release machinery. *Annu. Rev. Biophys.* 44 339–367. 10.1146/annurev-biophys-060414-034057 26098518

[B158] Roberts J. L. Hovanes K. Dasouki M. Manzardo A. M. Butler M. G. (2014). Chromosomal microarray analysis of consecutive individuals with autism spectrum disorders or learning disability presenting for genetic services. *Gene* 535 70–78. 10.1016/j.gene.2013.10.020 24188901 PMC4423794

[B159] Ronesi J. A. Collins K. A. Hays S. A. Tsai N. P. Guo W. Birnbaum S. G. (2012). Disrupted Homer scaffolds mediate abnormal mGluR5 function in a mouse model of fragile X syndrome. *Nat. Neurosci* 15 431–440. 10.1038/nn.3033 22267161 PMC3288402

[B160] Rotaru D. C. van Woerden G. M. Wallaard I. Elgersma Y. (2018). Adult Ube3a gene reinstatement restores the electrophysiological deficits of prefrontal cortex layer 5 neurons in a mouse model of angelman syndrome. *J. Neurosci.* 38 8011–8030. 10.1523/jneurosci.0083-18.2018 30082419 PMC6596147

[B161] Sandin S. Hultman C. M. Kolevzon A. Gross R. MacCabe J. H. Reichenberg A. (2012). Advancing maternal age is associated with increasing risk for autism: a review and meta-analysis. *J. Am. Acad. Child Adolesc. Psychiatry* 51 477–486.e471. 10.1016/j.jaac.2012.02.018 22525954

[B162] Sandin S. Lichtenstein P. Kuja-Halkola R. Larsson H. Hultman C. M. Reichenberg A. (2014). The familial risk of autism. *JAMA* 311 1770–1777. 10.1001/jama.2014.4144 24794370 PMC4381277

[B163] Sato D. Lionel A. C. Leblond C. S. Prasad A. Pinto D. Walker S. (2012). SHANK1 deletions in males with autism spectrum disorder. *Am. J. Hum. Genet.* 90 879–887. 10.1016/j.ajhg.2012.03.017 22503632 PMC3376495

[B164] Schmeisser M. J. Ey E. Wegener S. Bockmann J. Stempel A. V. Kuebler A. (2012). Autistic-like behaviours and hyperactivity in mice lacking ProSAP1/Shank2. *Nature* 486 256–260. 10.1038/nature11015 22699619

[B165] Schoch H. Kreibich A. S. Ferri S. L. White R. S. Bohorquez D. Banerjee A. (2017). Sociability deficits and altered amygdala circuits in mice lacking pcdh10, an autism associated gene. *Biol. Psychiatry* 81 193–202. 10.1016/j.biopsych.2016.06.008 27567313 PMC5161717

[B166] Shcheglovitov A. Shcheglovitova O. Yazawa M. Portmann T. Shu R. Sebastiano V. (2013). SHANK3 and IGF1 restore synaptic deficits in neurons from 22q13 deletion syndrome patients. *Nature* 503 267–271. 10.1038/nature12618 24132240 PMC5559273

[B167] Silverman J. L. Turner S. M. Barkan C. L. Tolu S. S. Saxena R. Hung A. Y. (2011). Sociability and motor functions in Shank1 mutant mice. *Brain Res.* 1380 120–137. 10.1016/j.brainres.2010.09.026 20868654 PMC3041833

[B168] Smith S. E. Zhou Y. D. Zhang G. Jin Z. Stoppel D. C. Anderson M. P. (2011). Increased gene dosage of Ube3a results in autism traits and decreased glutamate synaptic transmission in mice. *Sci. Transl. Med.* 3:103ra197. 10.1126/scitranslmed.3002627 21974935 PMC3356696

[B169] Song J. Y. Ichtchenko K. Sudhof T. C. Brose N. (1999). Neuroligin 1 is a postsynaptic cell-adhesion molecule of excitatory synapses. *Proc. Natl. Acad. Sci. U.S.A.* 96 1100–1105. 9927700 10.1073/pnas.96.3.1100 PMC15357

[B170] Sperow M. Berry R. B. Bayazitov I. T. Zhu G. Baker S. J. Zakharenko S. S. (2012). Phosphatase and tensin homologue (PTEN) regulates synaptic plasticity independently of its effect on neuronal morphology and migration. *J. Physiol.* 590 777–792. 10.1113/jphysiol.2011.220236 22147265 PMC3381310

[B171] Spinelli L. Black F. M. Berg J. N. Eickholt B. J. Leslie N. R. (2015). Functionally distinct groups of inherited PTEN mutations in autism and tumour syndromes. *J. Med. Genet.* 52 128–134. 10.1136/jmedgenet-2014-102803 25527629 PMC4316932

[B172] Stefanovic S. Bassell G. J. Mihailescu M. R. (2015). G quadruplex RNA structures in PSD-95 mRNA: potential regulators of miR-125a seed binding site accessibility. *RNA* 21 48–60. 10.1261/rna.046722.114 25406362 PMC4274637

[B173] Steffenburg S. Gillberg C. Hellgren L. Andersson L. Gillberg I. C. Jakobsson G. (1989). A twin study of autism in Denmark, Finland, Iceland, Norway and Sweden. *J. Child Psychol. Psychiatry* 30 405–416. 2745591 10.1111/j.1469-7610.1989.tb00254.x

[B174] Stessman H. A. Xiong B. Coe B. P. Wang T. Hoekzema K. Fenckova M. (2017). Targeted sequencing identifies 91 neurodevelopmental-disorder risk genes with autism and developmental-disability biases. *Nat. Genet.* 49 515–526. 10.1038/ng.3792 28191889 PMC5374041

[B175] Sudhof T. C. (2017). Synaptic neurexin complexes: a molecular code for the logic of neural circuits. *Cell* 171 745–769. 10.1016/j.cell.2017.10.024 29100073 PMC5694349

[B176] Sun J. Zhu G. Liu Y. Standley S. Ji A. Tunuguntla R. (2015). UBE3A regulates synaptic plasticity and learning and memory by controlling SK2 channel endocytosis. *Cell Rep.* 12 449–461. 10.1016/j.celrep.2015.06.023 26166566 PMC4520703

[B177] Sundberg M. Tochitsky I. Buchholz D. E. Winden K. Kujala V. Kapur K. (2018). Purkinje cells derived from TSC patients display hypoexcitability and synaptic deficits associated with reduced FMRP levels and reversed by rapamycin. *Mol. Psychiatry* 10.1038/s41380-018-0018-4 [Epub ahead of print]. 29449635 PMC6093816

[B178] Sungur A. O. Jochner M. C. E. Harb H. Kilic A. Garn H. Schwarting R. K. W. (2017). Aberrant cognitive phenotypes and altered hippocampal BDNF expression related to epigenetic modifications in mice lacking the post-synaptic scaffolding protein SHANK1: implications for autism spectrum disorder. *Hippocampus* 27 906–919. 10.1002/hipo.22741 28500650

[B179] Sungur A. O. Schwarting R. K. Wohr M. (2016). Early communication deficits in the Shank1 knockout mouse model for autism spectrum disorder: developmental aspects and effects of social context. *Autism Res.* 9 696–709. 10.1002/aur.1564 26419918

[B180] Sungur A. O. Vorckel K. J. Schwarting R. K. Wohr M. (2014). Repetitive behaviors in the Shank1 knockout mouse model for autism spectrum disorder: developmental aspects and effects of social context. *J. Neurosci. Methods* 234 92–100. 10.1016/j.jneumeth.2014.05.003 24820912

[B181] Tabuchi K. Blundell J. Etherton M. Hammer R. Liu X. Powell C. (2007). A neuroligin-3 mutation implicated in autism increases inhibitory synaptic transmission in mice. *Science* 318 71–76. 17823315 10.1126/science.1146221 PMC3235367

[B182] Taft C. E. Turrigiano G. G. (2014). PSD-95 promotes the stabilization of young synaptic contacts. *Philos. Trans. R. Soc. Lond. B Biol. Sci.* 369:20130134. 10.1098/rstb.2013.0134 24298137 PMC3843867

[B183] Takeuchi K. Gertner M. J. Zhou J. Parada L. F. Bennett M. V. Zukin R. S. (2013). Dysregulation of synaptic plasticity precedes appearance of morphological defects in a Pten conditional knockout mouse model of autism. *Proc. Natl. Acad. Sci. U.S.A.* 110 4738–4743. 10.1073/pnas.1222803110 23487788 PMC3607034

[B184] Tang B. Wang T. Wan H. Han L. Qin X. Zhang Y. (2015). Fmr1 deficiency promotes age-dependent alterations in the cortical synaptic proteome. *Proc. Natl. Acad. Sci. U.S.A.* 112 E4697–E4706. 10.1073/pnas.1502258112 26307763 PMC4553823

[B185] Tian Y. Yang C. Shang S. Cai Y. Deng X. Zhang J. (2017). Loss of FMRP impaired hippocampal long-term plasticity and spatial learning in rats. *Front. Mol. Neurosci.* 10:269. 10.3389/fnmol.2017.00269 28894415 PMC5581399

[B186] Till S. M. Asiminas A. Jackson A. D. Katsanevaki D. Barnes S. A. Osterweil E. K. (2015). Conserved hippocampal cellular pathophysiology but distinct behavioural deficits in a new rat model of FXS. *Hum. Mol. Genet.* 24 5977–5984. 10.1093/hmg/ddv299 26243794 PMC4599667

[B187] Toft A. K. Lundbye C. J. Banke T. G. (2016). Dysregulated NMDA-receptor signaling inhibits long-term depression in a mouse model of fragile X syndrome. *J. Neurosci.* 36 9817–9827. 10.1523/jneurosci.3038-15.2016 27656021 PMC6705565

[B188] Tsai N. Wilkerson J. Guo W. Huber K. (2017). FMRP-dependent Mdm2 dephosphorylation is required for MEF2-induced synapse elimination. *Hum. Mol. Genet.* 26 293–304. 10.1093/hmg/ddw386 28025327 PMC6075181

[B189] Tsai N. Wilkerson J. Guo W. Maksimova M. DeMartino G. Cowan C. (2012). Multiple autism-linked genes mediate synapse elimination via proteasomal degradation of a synaptic scaffold PSD-95. *Cell* 151 1581–1594. 10.1016/j.cell.2012.11.040 23260144 PMC3530171

[B190] Tsai P. T. Hull C. Chu Y. Greene-Colozzi E. Sadowski A. R. Leech J. M. (2012). Autistic-like behaviour and cerebellar dysfunction in Purkinje cell Tsc1 mutant mice. *Nature* 488 647–651. 10.1038/nature11310 22763451 PMC3615424

[B191] Tuchman R. Cuccaro M. Alessandri M. (2010). Autism and epilepsy: historical perspective. *Brain Dev.* 32 709–718. 10.1016/j.braindev.2010.04.008 20510557

[B192] Tyagarajan S. K. Ghosh H. Yevenes G. E. Imanishi S. Y. Zeilhofer H. U. Gerrits B. (2013). Extracellular signal-regulated kinase and glycogen synthase kinase 3beta regulate gephyrin postsynaptic aggregation and GABAergic synaptic function in a calpain-dependent mechanism. *J. Biol. Chem.* 288 9634–9647. 10.1074/jbc.M112.442616 23408424 PMC3617267

[B193] Tyagarajan S. K. Ghosh H. Yevenes G. E. Nikonenko I. Ebeling C. Schwerdel C. (2011). Regulation of GABAergic synapse formation and plasticity by GSK3beta-dependent phosphorylation of gephyrin. *Proc. Natl. Acad. Sci. U.S.A.* 108 379–384. 10.1073/pnas.1011824108 21173228 PMC3017200

[B194] Uddin M. Woodbury-Smith M. Chan A. Brunga L. Lamoureux S. Pellecchia G. (2017). Germline and somatic mutations in STXBP1 with diverse neurodevelopmental phenotypes. *Neurol Genet.* 3:e199. 10.1212/nxg.0000000000000199 29264391 PMC5735305

[B195] Vaags A. K. Lionel A. C. Sato D. Goodenberger M. Stein Q. P. Curran S. (2012). Rare deletions at the neurexin 3 locus in autism spectrum disorder. *Am. J. Hum. Genet.* 90 133–141. 10.1016/j.ajhg.2011.11.025 22209245 PMC3257896

[B196] van Woerden G. M. Harris K. D. Hojjati M. R. Gustin R. M. Qiu S. de Avila Freire R. (2007). Rescue of neurological deficits in a mouse model for Angelman syndrome by reduction of alphaCaMKII inhibitory phosphorylation. *Nat. Neurosci.* 10 280–282. 10.1038/nn1845 17259980

[B197] Varga E. A. Pastore M. Prior T. Herman G. E. McBride K. L. (2009). The prevalence of PTEN mutations in a clinical pediatric cohort with autism spectrum disorders, developmental delay, and macrocephaly. *Genet. Med.* 11 111–117. 10.1097/GIM.0b013e31818fd762 19265751

[B198] Varoqueaux F. Aramuni G. Rawson R. L. Mohrmann R. Missler M. Gottmann K. (2006). Neuroligins determine synapse maturation and function. *Neuron* 51 741–754. 10.1016/j.neuron.2006.09.003 16982420

[B199] Varoqueaux F. Jamain S. Brose N. (2004). Neuroligin 2 is exclusively localized to inhibitory synapses. *Eur. J. Cell Biol.* 83 449–456. 10.1078/0171-9335-00410 15540461

[B200] Vignoli A. La Briola F. Peron A. Turner K. Vannicola C. Saccani M. (2015). Autism spectrum disorder in tuberous sclerosis complex: searching for risk markers. *Orphanet J. Rare Dis.* 10:154. 10.1186/s13023-015-0371-1 26631248 PMC4668631

[B201] von der Brelie C. Waltereit R. Zhang L. Beck H. Kirschstein T. (2006). Impaired synaptic plasticity in a rat model of tuberous sclerosis. *Eur. J. Neurosci.* 23 686–692. 10.1111/j.1460-9568.2006.04594.x 16487150

[B202] Wallace M. L. Burette A. C. Weinberg R. J. Philpot B. D. (2012). Maternal loss of Ube3a produces an excitatory/inhibitory imbalance through neuron type-specific synaptic defects. *Neuron* 74 793–800. 10.1016/j.neuron.2012.03.036 22681684 PMC3372864

[B203] Wang C. Y. Lin H. C. Song Y. P. Hsu Y. T. Lin S. Y. Hsu P. C. (2015). Protein kinase C-dependent growth-associated protein 43 phosphorylation regulates gephyrin aggregation at developing GABAergic synapses. *Mol. Cell. Biol.* 35 1712–1726. 10.1128/mcb.01332-14 25755278 PMC4405633

[B204] Wang J. Li L. Shao S. S. He Z. Chen Y. L. Kong R. (2016). Association analysis of genetic variant of rs13331 in PSD95 gene with autism spectrum disorders: a case-control study in a Chinese population. *J. Huazhong Univ. Sci. Technolog. Med. Sci.* 36 285–288. 10.1007/s11596-016-1581-z 27072977

[B205] Wang T. Guo H. Xiong B. Stessman H. A. Wu H. Coe B. P. (2016). De novo genic mutations among a Chinese autism spectrum disorder cohort. *Nat. Commun.* 7:13316. 10.1038/ncomms13316 27824329 PMC5105161

[B206] Wang X. McCoy P. A. Rodriguiz R. M. Pan Y. Je H. S. Roberts A. C. (2011). Synaptic dysfunction and abnormal behaviors in mice lacking major isoforms of Shank3. *Hum. Mol. Genet.* 20 3093–3108. 10.1093/hmg/ddr212 21558424 PMC3131048

[B207] Wegener S. Buschler A. Stempel A. V. Kang S. J. Lim C. S. Kaang B. K. (2018). Defective synapse maturation and enhanced synaptic plasticity in Shank2 Deltaex7(-/-) Mice. *eNeuro* 5:ENEURO.0398-17.2018. 10.1523/eneuro.0398-17.2018 30023428 PMC6049608

[B208] Weng S. M. McLeod F. Bailey M. E. Cobb S. R. (2011). Synaptic plasticity deficits in an experimental model of rett syndrome: long-term potentiation saturation and its pharmacological reversal. *Neuroscience* 180 314–321. 10.1016/j.neuroscience.2011.01.061 21296130

[B209] Weston M. C. Chen H. Swann J. W. (2012). Multiple roles for mammalian target of rapamycin signaling in both glutamatergic and GABAergic synaptic transmission. *J. Neurosci.* 32 11441–11452. 10.1523/jneurosci.1283-12.2012 22895726 PMC3432956

[B210] Wilkerson J. R. Tsai N. P. Maksimova M. A. Wu H. Cabalo N. P. Loerwald K. W. (2014). A role for dendritic mGluR5-mediated local translation of Arc/Arg3.1 in MEF2-dependent synapse elimination. *Cell Rep.* 7 1589–1600. 10.1016/j.celrep.2014.04.035 24857654 PMC4057996

[B211] Williams M. R. DeSpenza T. Jr. Li M. Gulledge A. T. Luikart B. W. (2015). Hyperactivity of newborn Pten knock-out neurons results from increased excitatory synaptic drive. *J. Neurosci.* 35 943–959. 10.1523/jneurosci.3144-14.2015 25609613 PMC4300333

[B212] Wohr M. Roullet F. I. Hung A. Y. Sheng M. Crawley J. N. (2011). Communication impairments in mice lacking Shank1: reduced levels of ultrasonic vocalizations and scent marking behavior. *PLoS One* 6:e20631. 10.1371/journal.pone.0020631 21695253 PMC3111434

[B213] Won H. Lee H. R. Gee H. Y. Mah W. Kim J. I. Lee J. (2012). Autistic-like social behaviour in Shank2-mutant mice improved by restoring NMDA receptor function. *Nature* 486 261–265. 10.1038/nature11208 22699620

[B214] Xing G. Li M. Sun Y. Rui M. Zhuang Y. Lv H. (2018). Neurexin-Neuroligin 1 regulates synaptic morphology and functions via the WAVE regulatory complex in Drosophila neuromuscular junction. *Elife* 7:e30457. 10.7554/eLife.30457 29537369 PMC5873926

[B215] Xing J. Kimura H. Wang C. Ishizuka K. Kushima I. Arioka Y. (2016). Resequencing and association analysis of Six PSD-95-related genes as possible susceptibility genes for schizophrenia and autism spectrum disorders. *Sci. Rep.* 6:27491. 10.1038/srep27491 27271353 PMC4895433

[B216] Xu X. Li C. Gao X. Xia K. Guo H. Li Y. (2018). Excessive UBE3A dosage impairs retinoic acid signaling and synaptic plasticity in autism spectrum disorders. *Cell Res.* 28 48–68. 10.1038/cr.2017.132 29076503 PMC5752837

[B217] Yang Y. M. Arsenault J. Bah A. Krzeminski M. Fekete A. Chao O. Y. (2018). Identification of a molecular locus for normalizing dysregulated GABA release from interneurons in the Fragile X brain. *Mol. Psychiatry.* 10.1038/s41380-018-0240-0 [Epub ahead of print]. 30224722 PMC7473840

[B218] Yangngam S. Plong-On O. Sripo T. Roongpraiwan R. Hansakunachai T. Wirojanan J. (2014). Mutation screening of the neurexin 1 gene in thai patients with intellectual disability and autism spectrum disorder. *Genet. Test Mol. Biomarkers* 18 510–515. 10.1089/gtmb.2014.0003 24832020 PMC4094023

[B219] Yuan H. Wang Q. Liu Y. Yang W. He Y. Gusella J. F. (2018). A rare exonic NRXN3 deletion segregating with neurodevelopmental and neuropsychiatric conditions in a three-generation Chinese family. *Am. J. Med. Genet. B Neuropsychiatr. Genet.* 177 589–595. 10.1002/ajmg.b.32673 30076746 PMC6445570

[B220] Zhang Y. Gaetano C. M. Williams K. R. Bassell G. J. Mihailescu M. R. (2014). FMRP interacts with G-quadruplex structures in the 3’-UTR of its dendritic target Shank1 mRNA. *RNA Biol.* 11 1364–1374. 10.1080/15476286.2014.996464 25692235 PMC4615183

[B221] Ziats M. N. Rennert O. M. (2016). The evolving diagnostic and genetic landscapes of autism spectrum disorder. *Front. Genet.* 7:65. 10.3389/fgene.2016.00065 27200076 PMC4844926

